# Neuropeptidergic Systems in Pluteus Larvae of the Sea Urchin *Strongylocentrotus purpuratus*: Neurochemical Complexity in a “Simple” Nervous System

**DOI:** 10.3389/fendo.2018.00628

**Published:** 2018-10-25

**Authors:** Natalie J. Wood, Teresa Mattiello, Matthew L. Rowe, Lizzy Ward, Margherita Perillo, Maria Ina Arnone, Maurice R. Elphick, Paola Oliveri

**Affiliations:** ^1^Centre for Life's Origins and Evolution, Research Department of Genetics, Evolution and Environment, University College London, London, United Kingdom; ^2^Stazione Zoologica Anton Dohrn, Naples, Italy; ^3^School of Biological and Chemical Sciences, Queen Mary University of London, London, United Kingdom; ^4^Research Department of Cell and Developmental Biology, University College London, London, United Kingdom

**Keywords:** neuron, co-expression, neuropeptide, echinoderm, embryo, serotonin

## Abstract

The nervous system of the free-living planktonic larvae of sea urchins is relatively “simple,” but sufficiently complex to enable sensing of the environment and control of swimming and feeding behaviors. At the pluteus stage of development, the nervous system comprises a central ganglion of serotonergic neurons located in the apical organ and sensory and motor neurons associated with the ciliary band and the gut. Neuropeptides are key mediators of neuronal signaling in nervous systems but currently little is known about neuropeptidergic systems in sea urchin larvae. Analysis of the genome sequence of the sea urchin *Strongylocentrotus purpuratus* has enabled the identification of 38 genes encoding neuropeptide precursors (NP) in this species. Here we characterize for the first time the expression of nine of these NP genes in *S. purpuratus* larvae, providing a basis for a functional understanding of the neurochemical organization of the larval nervous system. In order to accomplish this we used single and double *in situ* hybridization, coupled with immunohistochemistry, to investigate NP gene expression in comparison with known markers (e.g., the neurotransmitter serotonin). Several sub-populations of cells that express one or more NP genes were identified, which are located in the apica organ, at the base of the arms, around the mouth, in the ciliary band and in the mid- and fore-gut. Furthermore, high levels of cell proliferation were observed in neurogenic territories, consistent with an increase in the number of neuropeptidergic cells at late larval stages. This study has revealed that the sea urchin larval nervous system is far more complex at a neurochemical level than was previously known. Our NP gene expression map provides the basis for future work, aimed at understanding the role of diverse neuropeptides in control of various aspects of embryonic and larval behavior.

## Introduction

The evolution of neuronal cell types and nervous systems is a hotly debated topic (1, 2), reflecting the diversity in nervous system organization found in animals. Understanding the complexity and organization of neuronal cell types in less studied taxa may help to provide new insights into neural evolution. As invertebrate deuterostomes, the echinoderms (sea urchins, sea stars, brittle stars, sea cucumbers, and feather stars) are a key phylum for evolutionary studies because they “bridge the gap” between the intensely studied vertebrates and protostomian model invertebrates such as *Drosophila* and *C. elegans*.

Echinoderm embryos develop into free-living planktonic larvae equipped with a relatively “simple” nervous system, typified by the sea urchin pluteus larva, that allows them to sense the environment and to control swimming and feeding behaviors. The larval nervous system of sea urchins is thought to be completely independent of the adult nervous system, which develops in the larval rudiment (3). The sea urchin larva, therefore, offers opportunities to observe the complete development and function of a nervous system in a small and tractable organism. The larval nervous system is the “product” of neurogenic processes that have been studied using immunostaining for neural markers and analysis of the expression of pro-neuronal genes, as reviewed in Hinman and Burke (4). However, the diversity of neuronal sub-types in sea urchin larvae has not been characterized in detail.

Neurogenic capacity in the sea urchin embryo is initially present throughout the entire ectoderm, but later it becomes restricted to the anterior neuroectoderm (ANE) and the ciliary band neuroectoderm (CBE), under the control of Wnt-dependent pathways that regulate Nodal and bone morphogenetic protein (BMP) signaling (5). The first neurons, sensory serotonergic neurons, appear in the apical plate at the gastrula stage (48 hours post-fertilization (hpf) in *S. purpuratus*), along with two cells in the oral ectoderm that will develop into the post-oral neurons (6). From here onwards, the nervous system continues to grow and become increasingly complex as the larva increases in size from a four-arm pluteus to an eight-arm competent larva (6–8). Pioneering electron microscopy (EM) studies and more recent use of pan-neural markers (e.g. synaptotagmin) have provided a detailed picture of the number of neurons and the organization of the larval nervous system in the apical organ, which is considered the central nervous system of the larva, whereas the ciliary band and the lateral ganglia are considered the peripheral nervous system (3, 4, 6, 9)]. Populations of neurons, formed from neuro-endoderm precursor cells, have also been identified in the pharynx (10). The identification of sub-populations of larval neurons has thus far been largely restricted to the analysis of the neurotransmitters serotonin, dopamine and GABA (6). In the early four-arm larva, Bisgrove and Burke identified serotonin-positive neurons in apical organ cells, dopamine-positive neurons in the lower lip and post-oral cells, and GABA-positive neurons in the esophagus.

A major class of neuromodulators in the nervous system are neuropeptides, which are ancient intercellular signaling molecules that act through specific G protein-coupled receptors (GPCRs) to mediate neuronal regulation of many physiological and behavioral processes. Neuropeptides are present across the Eumetazoa, suggesting that these ancient molecules play a key role in the function and evolution of nervous systems (11, 12). The first neuropeptides to be identified in echinoderms were the starfish SALMFamides S1 and S2 (13, 14). Using antibodies to S1 and S2, Beer and collaborators produced the first description of a neuropeptidergic component of the larval sea urchin nervous system (8). However, the molecular identity of the immunoreactive molecules was unknown. Sequencing of the sea urchin genome has enabled the identification of genes encoding candidate secreted peptides in *S. purpuratus* (15), including genes encoding two types of SALMFamides - F-type SALMFamides, which have the C-terminal motif Phe-X-Phe-NH_2_, and L-type SALMFamides, which like S1 and S2 have the C-terminal motif (Leu/lle)-X-Phe-NH_2_ and which are presumably the neuropeptides that are recognized by antibodies to S1 and S2. Other neuropeptide precursor (NP) genes identified in the genome of *S. purpuratus* include genes encoding paralogous precursors of the vasopressin/oxytocin-type neuropeptide echinotocin and the neuropeptide NGFFFamide (15–17). Furthermore, a detailed analysis of cDNAs derived from a radial nerve cDNA library enabled the identification of 20 putative NP genes in *S. purpuratus*, including seven that share sequence similarity with known neuropeptides and 13 that were not found to share sequence similarity with known neuropeptides (18, 19). Six additional putative NP genes were identified in *S. purpuratus*, in parallel with the discovery of homologs in other echinoderms (starfish *Asterias rubens*, brittle stars *Ophionotus victoriae, Amphiura filiformis*, and *Ophiopsila aranea*) and also through phylogenomic studies (20–23). Mass spectrometry has also been used to determine the structures of some of the neuropeptides encoded by NP genes in *S. purpuratus* (16, 18, 24, 25). Furthermore, characterization of neuropeptides and neuropeptide receptors in *S. purpuratus* and other echinoderms has provided important insights into the evolution of neuropeptide signaling. For example, discovery of the receptor for the neuropeptide NGFFFamide in *S. purpuratus* facilitated the reconstruction of the common evolutionary history of neuropeptide-S-type signaling in vertebrates and crustacean cardioactive peptide (CCAP)-type signaling in protostomes (16). Secreted peptide signaling molecules have also been identified in association with the larval sea urchin gut. Perillo and Arnone (26) reported specific cells in the anterior region of the gut that express a *Sp-Insulin-like peptide 1* (*Sp-ILP1*) NP gene together with other molecular markers typical of pancreatic exocrine-like cell types. Furthermore, expression of *Sp-ILP1* in the gut is affected by different feeding regimes (26), highlighting an ancient deuterostome role of ILP secreted peptides and the power of echinoderms in helping resolve evolutionary questions. Against this background, there now exists the opportunity to investigate the expression of multiple NP genes in populations of neurons in larval sea urchins, and to correlate findings with existing knowledge of the larval nervous system.

Recently, the first multi-gene analysis of NP gene expression in echinoderm larvae was reported, with mRNA *in situ* hybridization employed to analyse the expression of eight NP genes in the starfish *Asterias rubens* (27). Here we describe the complement of NP genes in the *S. purpuratus* genome, the temporal expression of 31 NP genes and the spatial expression of nine NP genes during larval development of the sea urchin *S. purpuratus*, using Quantitative PCR (QPCR) and mRNA *in situ* hybridization (ISH), respectively. Then having compared the patterns of expression, we have used double-labeling techniques to investigate NP gene expression in comparison with markers for other neurotransmitters (e.g., serotonin). The identification of specific populations of cells, neurons, and gut cells expressing NP genes enriches our understanding of the diversity of neuronal cell types in sea urchin larvae and the complexity of the larval nervous system.

## Methods

### Animal husbandry and embryonic and larval culture

Adult specimens of the purple sea urchin *Strongylocentrotus purpuratus* were obtained from Patrick Leahy (Kerchoff Marine Laboratory, California Institute of Technology, Pasadena, CA, USA) and housed in closed seawater aquaria at University College London and Stazione Zoologica Anton Dohrn of Naples at 14°C. Gametes and embryos were obtained from *S. purpuratus* and cultured as previously described (28). Filtered artificial seawater (FASW; 34.6ppt salinity) containing the antibiotics streptomycin (50 μg/mL) and penicillin (20 U/mL) was used as an alternative to seawater for maintenance of embryos.

### Isolation of cDNAs encoding NPs

Clones of cDNAs encoding NP genes were obtained from an *S. purpuratus* radial nerve arrayed cDNA library (Caltech; LIBEST_019872). Fragments of cDNAs encoding *Sp-Kp, Sp-Np20* and *Sp-Nesf* were amplified from cDNA obtained from embryos/larvae at 18, 21, and 27 hpf, and 1-week, and then cloned in a pGEM-T® easy vector system (Promega) according to the manufacturer's instructions. Antisense probes were synthesized after sequencing. Primers and probe information are presented in Supplementary Table 1.

### *In situ* hybridization (ISH)

Embryo and larvae were fixed as previously described (29). For single fluorescent or chromogenic whole-mount ISH, antisense probes were transcribed from isolated cDNA fragments and labeled using 10X DIG RNA labeling mix (Roche). For double fluorescent ISH, digoxigenin-11-UTP (Roche), and Label IT® DNP (Mirus, MIR3800) antisense probes were synthesized, according to the manufacturer's protocol. For both single and double fluorescent ISH experiments, probes were used at a final concentration of 0.03–0.05 ng/μL.

For a detailed ISH procedure, see Supplementary Material. Nuclei were stained in 2.5 μg/mL DAPI in PBST. At least three embryos were imaged in detail by confocal microscopy and others by fluorescent microscopy using three-channel fluorescence and DIC images were generated using a Zeiss 510 Meta Light Microscope or a Leica TCS SPE2. Image analysis was done using FIJI and Adobe Photoshop software. A developmental stage that was known not to express a particular gene was used as a negative control. For chromogenic ISH *Sp-FoxA* was used as a positive control as the spatial expression pattern is well characterized across development (30). Single chromogenic ISH was first completed for all genes later analyzed using double fluorescent ISH.

### Immunostaining

Embryo and larvae were fixed in 4% paraformaldehyde. Rabbit polyclonal anti-serotonin (Sigma) and mouse anti-acetylated tubulin (Sigma), mouse anti-synaptotagminB (SynB/1E11) (31) were used. Secondary antibodies Alexa 488 goat anti-rabbit, Alexa 633 donkey anti-mouse and Alexa 488 goat anti-mouse (Thermo Scientifics) were used at a dilution of 1:250. For a detailed protocol, see Supplementary Material.

### Edu labeling

Larvae at 4 days of development were incubated in ethynyl deoxyuridine (EdU) for 2 h using the Click-iT™ EdU Alexa Fluor™ 555 Imaging Kit (ThermoFisher), according to the manufacturer's instructions (for details see Supplementary Material).

### Quantitative PCR (QPCR)

Total RNA was isolated from batches of embryos at different stages of development. The RNA was extracted using the RNAeasy Micro Kit (Qiagen), according to the manufacturer's instructions. First-strand cDNA was synthesized using a maximum of 1 μg of total RNA and the iScript™ cDNA synthesis kit (Bio-Rad), as described by the manufacturer. The cDNA was diluted to 2.8 ng/μl and used directly for quantitative PCR (QPCR) analysis. QPCR was conducted as previously described (32) and each sample was run in quadruplicate technical replicas. For QPCR experiments, the data from each cDNA sample (0–72 hpf) were normalized against ubiquitin mRNA levels, which are known to remain relatively constant during development (33–35). cDNA samples from 5 week old larvae and juveniles were normalized against 18S rRNA and compared using relative expression (individual maximum expression). cDNA was substituted with water for negative control experiments. The primers used can be found in Supplementary Table 2.

## Results

### The complexity of the neuropeptidome in the developing sea urchin larvae

To obtain a detailed overview of NP gene expression in the developing nervous system of the sea urchin embryo and larva, we first analyzed *S. purpuratus* genome sequence data to characterize the complexity of the neuropeptidome in this species. Previous studies (18, 20, 21, 24) have identified 34 NP genes belonging to over 20 different families. Of these genes, 14 NPs were found not to share sequence similarity with known NPs from other species (18). The availability of genome (36) and transcriptome (22, 23) sequence data from other echinoderms enabled the identification of a total of 38 NP genes, including the previously identified genes. The latest version of the *S. purpuratus* genome (Echinobase, v 4.2; http://www.echinobase.org/Echinobase/) was analyzed using newly identified echinoderm NP sequences as queries for BlastP searches and SignalP (http://www.cbs.dtu.dk/services/SignalP-3.0/; (37)) was used to determine the presence of a signal peptide, which is a characteristic feature of neuropeptide precursors and other secreted proteins. This analysis enabled identification of *S. purpuratus* NP genes that have been reported previously as well as the *Sp-Kp* gene. These findings were confirmed independently (22, 23).

**Figure 1:**
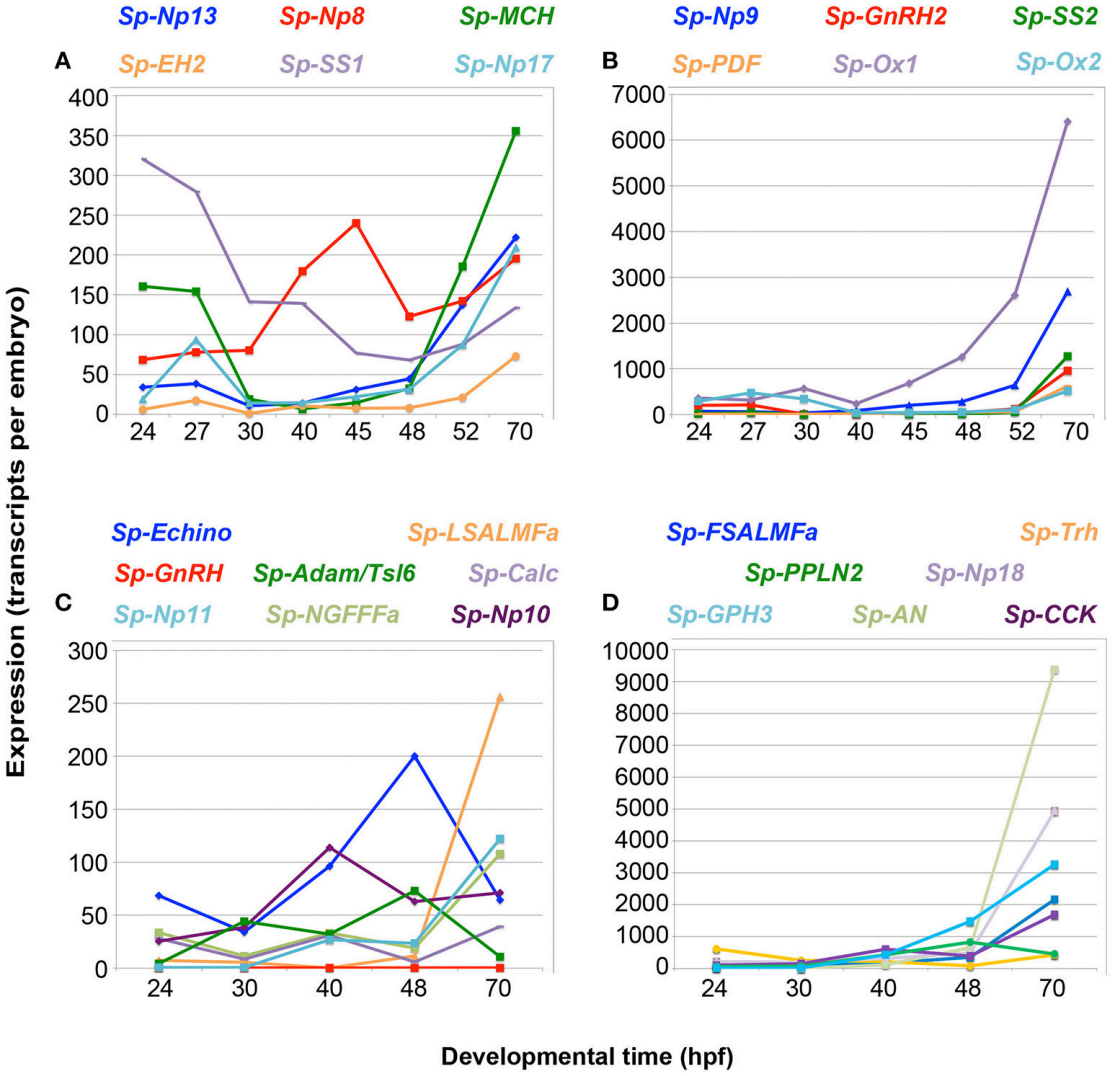
Quantitative PCR (QPCR) reveals that NP gene expression correlates with an increase in the complexity of the larval nervous system. NP gene expression (transcripts per embryo) is shown from the appearance of the first neuronal precursor cells at mesenchyme blastula stage [24 h post-fertilization (hpf)], through embryonic development to the early larval stage (70 hpf) when several types of neurons are differentiated. An increase in expression is seen for most NP genes from 48 to 70 hpf, when the first serotonergic neurons differentiate. The line graphs depict different levels of gene expression for 27 NP genes, with lower expression in graphs **(A,C)** and higher expression in graphs **(B,D)**. The color of the NP gene name above each graph reflects the line color in the corresponding graph.

Once the complement of NP genes present in the sea urchin genome was established, we studied their temporal expression during the development of the sea urchin nervous system: from the appearance of the first neuronal precursor cells at mesenchyme blastula (24 hpf), through embryonic development to the early larval stage (72 hpf) when several types of neurons are differentiated (6, 9, 38, 39). For this we used the available data from a *S. purpuratus* quantitative developmental transcriptome [http://www.echinobase.org/Echinobase/; (40)]and we also carried out quantitative real-time PCR (QPCR) measurements for all the NP genes not annotated in the latest *S. purpuratus* genome (Echinobase, v4.2), but predicted by NCBI or transcriptome studies. To be directly comparable to the transcriptome data, QPCR time points were chosen only up to end of development (70 hpf). We also studied the expression of 27 NP genes randomly choosen in two postembryonic stages: 5-week-old larvae and the juvenile stage. Generally, NP transcripts are present at very low levels throughout mesenchyme blastula and early gastrula stages (Figure 1; Supplementary Table 3; Supplementary Figure 4B), with the total number of transcripts ranging between 50 and 300 transcripts per embryo. Transcript levels then steadily increase throughout the prism and pluteus stages (48–70 hpf), with the total number of transcripts ranging between 300 and 42,000 transcripts per embryo (Figure 1; Supplementary Table 3). There are three exceptions to this pattern: (i) The *Sp-Orexin 1* (*Sp-Ox1)* and *Sp-Orexin 2* (*Sp-Ox2)* genes have a high number of transcripts (>300 transcripts per embryo) during mesenchyme blastula or early gastrula stages (Figure 1B). (ii) The *Sp-Thyrotropin-releasing hormone* (*Sp-Trh*) gene has a high number of transcripts (>300 transcripts per embryo) during mesenchyme blastula and then has a reduction in expression during gastrula and prism stages, before the number of transcripts increase to 426 transcripts per embryo at 70 hpf (Figure 1D). (iii) The *Sp-Somatostatin 1* (*Sp-SS1*) gene has a peak in expression at the mesenchyme blastula stage (>300 transcripts per embryo) followed by a gradual decrease in transcript levels for the remaining embryonic stages (Figure 1A). The QPCR data were in agreement with transcriptome data available from Echinobase (40) (Supplementary Figure 4). Furthermore, these two techniques quantified gene expression in different batches of embryos, thus validating the overall expression trends and the absolute number of transcripts per embryos. The increase in expression at 48 hpf, seen in most NP genes (23 out of 31), is coincident with the onset of neural differentiation (31, 41). Increase in expression at subsequent stages might reflect the increased number of neuronal precursors/neurons expressing NP genes.

Of the 38 NP genes identified in the sea urchin genome, 27 were analyzed for their expression: at a very early larval stage (70 hpf) when only the larval nervous system is present, which was used as a positive control; at the juvenile stage, when only the adult nervous system is present; and a late larval stage at 5 weeks (advanced rudiment stage), when both the larval and juvenile nervous systems are present. The temporal patterns of NP gene expression during post-embryonic stages are quite diverse (Supplementary Figure 5). The majority of NP genes, including *Sp-Trh* and *Sp-L-SALMFa*, among others (Supplementary Figures 5A,C), exhibit an increase in relative expression as larval development proceeds, with maximum expression at the juvenile stage. This trend correlates with an increase in the number of neuronal cells during the larval and juvenile stages. One group of NP genes, including *Sp-AN*, [containing an N-terminal alanine (A)/asparagine (N) (AN) motif] (Supplementary Figure 5B) also show a maximum expression at the juvenile stage, but have higher expression at the end of embryogenesis (70 hpf, pluteus stage) than in the 5 week old larvae. In contrast, *Sp-Glycoprotein hormone 3* (*Sp- GPH3)* and *Sp-Eclosion hormone 2 (Sp-EH2)* (Supplementary Figure 5D) have higher expression at the end of embryogenesis (70hpf, pluteus stage) and then their expression steadily decreases in later larval and juvenile stages. Collectively, these data show that NP gene expression largely correlates with the development of the sea urchin larval nervous system and the increased complexity in post-embryonic stages, suggesting that these genes are expressed in neurons.

### Early expression of neuropeptide precursors in neural precursor cells

To obtain more detailed insights into the expression of NP genes in the developing nervous system of sea urchin larvae, we next examined their spatial expression patterns at the gastrula stage (48 hpf) when the first neuronal precursor cells are detected and an overall increase in NP gene expression is evident (Figure 1) (39, 41). Two populations of neural precursor cells are present by the 48 hpf gastrula stage: serotonergic neural precursors in the apical plate and post-oral precursors in the oral ectoderm (39, 41). Double fluorescent ISH analysis was undertaken to determine the expression pattern of eight NP genes in the larval nervous system, relative to serotonin, a marker for apical plate neurons, and synaptotagmin, a pan-neuronal marker. In this work, for simplicity, we will refer to **neurons** if the co-expression of a NP gene with one or both of these two neuronal markers is reported, while we will refer to **neuron-like** cells, when the position and the shape of the NP-positive cell(s) is consistent with previously described neurons, but no expression with a neuronal differentiation marker is reported.

Out of the eight probes tested, five show clear staining at this stage, consistent with QPCR and transcriptome data at 48 hpf (Figures 1C,D; Supplementary Table 3). Indeed only the genes with expression levels above 300 transcripts/embryo are detected by ISH at this stage (Supplementary Table 3), while *Sp-Trh* and *Sp-Kisspeptin* (*Sp-Kp*), which both have <100 transcripts/embryo at 48 hpf, are not detected by this technique (data not shown). Transcriptome data available from Echinobase reveals that the *Sp-Kp* and *Sp-Np20* NP genes have low relative expression at 48 hpf (Supplementary Figure 3). This limit of detection is also supported by the absence of staining in WMISH experiments conducted with the same probes at stages with little or no expression (negative controls) and it is in agreement with published data (40). The exception is the *Sp-NGFFFamide* (*Sp-NGFFFa*) NP gene which has only 19 transcripts per embryo by our QPCR analysis and 47 transcripts per embryo by transcriptome quantification [Figure 1D; Supplementary Table 3; Supplementary Figure 4; (40)] and shows expression detectable by ISH. This could be explained by an under estimation of the level of expression of these quantitative techniques, or the highly localized expression in just a few cells.

**Figure 2:**
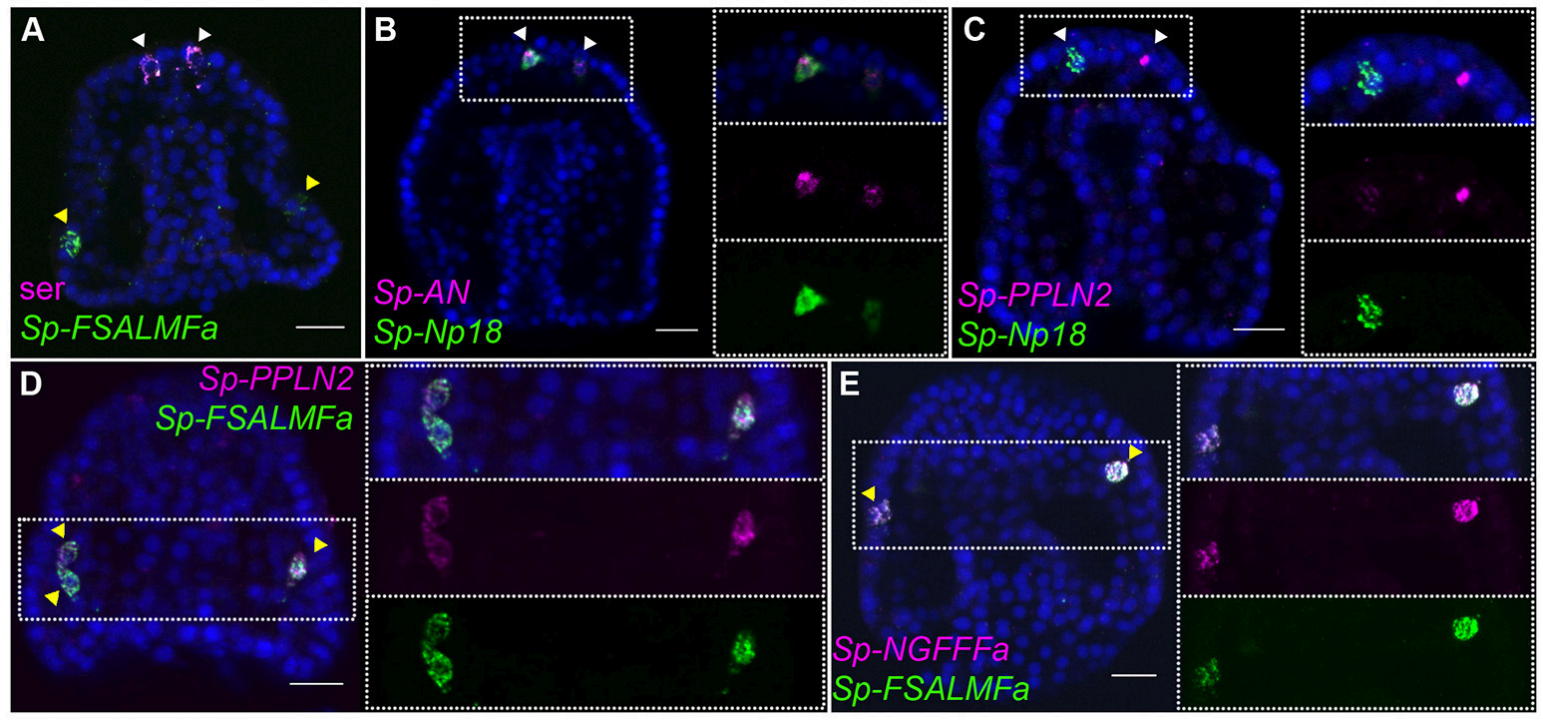
Expression of NP genes in the apical plate and post-oral neuron-like precursor cells of the gastrula embryo. Merged and single channel confocal images of 48 hpf embryos labeled using double fluorescent ISH are shown. **(A)** Expression of serotonin and *Sp-FSALMFa* highlights two populations present at 48 hpf, the serotonergic (white arrowheads) and post-oral (yellow arrowheads) neuron-like precursor cells, respectively. **(B,C)** Co-expression of three NP genes in the apical plate sensory precursors. **(D,E)** Co-expression of three NP genes in two-three post-oral neuron-like precursor cells. Bottom-left/Top-right corner indicates the probe or antibody used. Dotted white boxes highlight the magnified region shown to the right. Scale bars: 20 μm.

Co-expression of three NP genes, *Sp-AN, Sp-Np18*, and *Sp-Pedal peptide-like neuropeptide 2* (*Sp-PPLN2)*, is detected in two cells in the apical domain, which are also positive for serotonin and *Sp-SynB* (Figures 2A–C; Supplementary Figure 6A), whereas expression of the *Sp-FSALMFamide* (*Sp-FSALMFa*), *Sp-NGFFFa* and *Sp-PPLN2* NP genes is detected in one or two cells bilaterally arrayed in the oral ectoderm, in a position consistent with post-oral neuronal precursors [Figures 2A,D,E; (39, 41)].

In summary, spatial expression data revealed that various NP genes are expressed before the neurons are fully differentiated. Furthermore, distinct combinations of NP genes are co-expressed in specific populations of neuronal precursors at the gastrula stage.

### Expression of neuropeptide precursors in the larval nervous system

**Figure 3:**
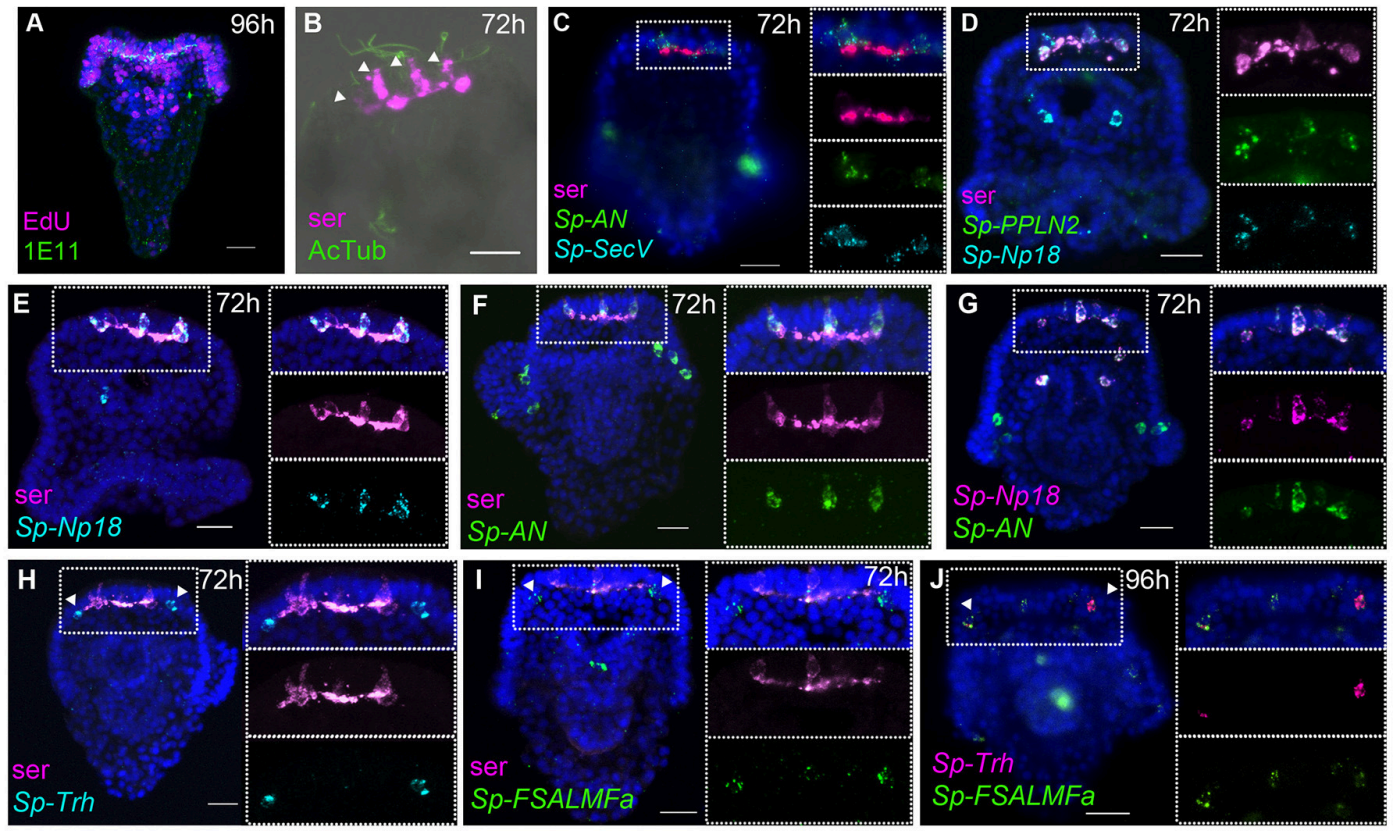
Expression of NP genes in the apical plate serotonergic neurons and ciliary band neuron-like cells. Merged and single channel confocal images of pluteus larvae labeled using double fluorescent ISH and immunohistochemistry are shown. **(A)** EdU-and SynB (1E11) labeling experiment showing a high rate of cell proliferation in the neurogenic territories (apical plate and ciliary band, lip and foregut). **(B,C)** Labeling of the larval nervous system using antibodies to serotonin and to acetylated tubulin (AcTub) and using *Sp-SecV* ISH. **(B–G)** NP gene expression in the apical plate serotonergic neurons. **(H–J)** *Sp-Trh* and *Sp-FSALMFa* co-expressed in the ciliary band, at the base of the oral distal arms, adjacent to the apical plate. Bottom-left corner indicates the probe or antibody used. Top-right corner indicates the larval stage in hours' post-fertilization; bottom-left corner indicates the probe or antibody used. Dotted white boxes highlight the magnified region shown to the right. Scale bars: 20 μm.

During post-gastrula development the number of neurons and neuronal precursor cells increases dramatically, consistent with the high level of proliferation detected by EdU staining, mainly in neurogenic tissues of the pluteus stage, such as the apical organ, ciliary band, and oral ectoderm (41). At the end of development, the simple nervous system of the pluteus larva (72 hpf) is composed of 40–50 neurons, which have differentiated in the apical organ [containing eight to nine serotonergic neurons (42)], ciliary band, lateral ganglia, post-oral, lip, and gut regions (6, 9, 10, 15, 38, 39, 41). The number of neurons and the complexity of the nervous system continues to increase during postembryonic larval development (6), consistent with the large number of proliferative cells identified by EdU staining located almost exclusively in neurogenic tissues of the pluteus (Figure 3A). To gain more detailed insights into the expression of NP genes relative to the known neuronal structures and the complexity of the larval nervous system, we studied the spatial expression of genes that have high levels of expression (Figure 1) using single probe chromogenic and double probe fluorescent ISH. Furthermore, we analyzed the expression of an ortholog of the human *SecretograninV (7B2L)* gene, *Sp-SecV*, a marker of neuroendocrine cells (43).

#### Central and peripheral nervous system

At the pluteus stage (72 hpf), the number of serotonergic neurons in the apical organ has increased and will continue to increase during larval development, consistent with the findings from EdU labeling, which illustrate a high rate of cell proliferation in the apical plate, ciliary band, lip, and foregut tissues (41) (Figure 3A). Furthermore, EdU labeling coupled with immunohistochemistry with the pan-neuronal marker SynB [1E11; (31)] revealed that neurons in the ciliary band are both mitotic (SynB^+^/Edu^+^) or post-mitotic (SynB^+^/EdU) (Supplementary Figure 6B). This is important to explain the different number of NP gene expressing cells identified in pluteus larvae.

The apical organ, considered the central nervous system of the sea urchin larva, consists of two types of serotonergic neurons: bottle shaped primary sensory neurons associated with long cilia of the apical tuft (Figure 3B) and small interconnected serotonergic neurons that form a ganglion (Figure 3), along with support cells (4). Fluorescent ISH (FISH) analysis of the expression of neuroendocrine marker *Sp-SecV* only labeled the primary sensory serotonergic neurons in the apical organ (Figure 3C), indicating a neuroendocrine role for these cells. Furthermore, various probe combinations in FISH experiments revealed that the *Sp-Np18, Sp-AN*, and *Sp-PPLN2* NP genes continue to be co-expressed in 2-5 cells with a morphology typical of sensory neurons that also express *Sp-SecV* and serotonin (Figures 3C–G; Supplementary Figure 6H). On either side, next to the serotonergic central neuronal system, two neuron-like cells at the base the oral distal arms (Figures 3H–J) were identified by the expression of the *Sp-Trh* and *Sp-FSALMFa* NP genes (Figures 3H–J). These cells are associated with the ciliary band and send projections toward the apical ganglion and their fibers intermingle with the fibers of the serotonergic interneurons, which is most clearly seen in 1-week old larvae (Supplementary Figure 6C). The cells do not express serotonin or *Sp-SecV*, suggesting they are a different population of neurons that communicate with the serotonergic central ganglion.

**Figure 4:**
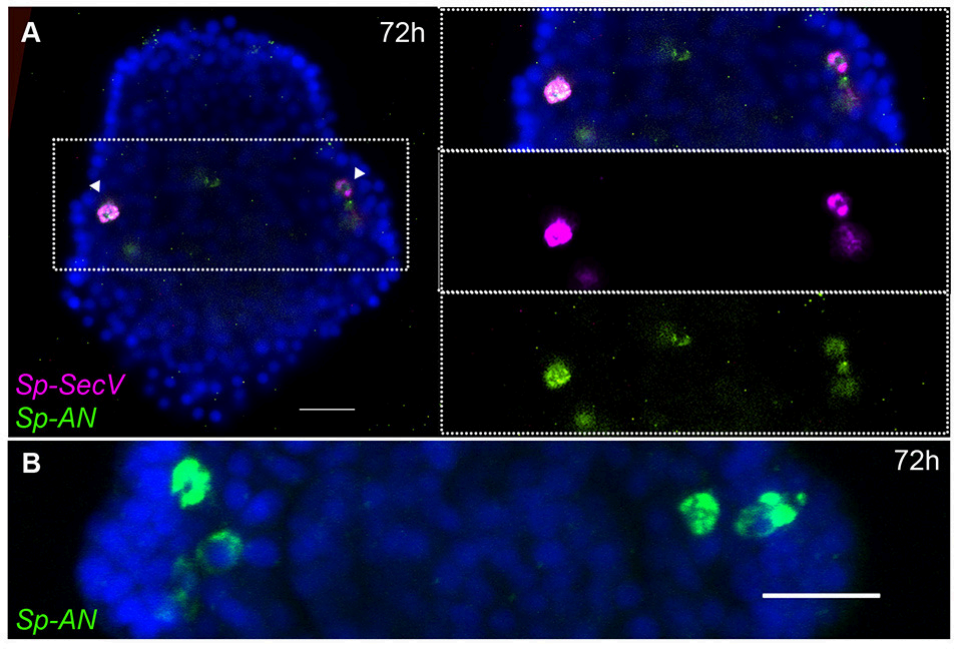
Expression of NP genes in two clusters of two-three lateral ganglia neuron-like cells. Merged confocal images of pluteus larvae labeled using single and double FISH are shown. **(A)** *Sp-SecV* and *Sp-AN* NP gene expression in lateral ganglia neuron-like cells. **(B)** Magnification of *Sp-AN* NP gene expression in the lateral ganglia neuron-like cells. Bottom-left corner indicates the probe used. Top-right corner indicates the larval stage in hours' post-fertilization. Dotted white boxes highlight the magnified region shown to the right. Scale bars: 20 μm.

The peripheral nervous system of the sea urchin larva is composed of neurons in the ciliary band, post-oral neurons, and lateral ganglion. *Sp-FSALMFa* is expressed in two single cells in the ciliary band at the base of the post-oral arms (Supplementary Figure 6D). The location is consistent with the expression of *Sp-FSALMFa* NP gene in the presumptive dopaminergic post-oral neurons (6, 44), and with the expression of this gene at the gastrula stage (Figures 2A,D,E). Interestingly, the expression of no other NP gene analyzed in this study was detected in these cells at the pluteus stage (Supplementary Figures 6A–E). The lateral ganglion neurons consist of two clusters of two-three neurons on the left and right side of the larva (3, 15) and are associated with the ciliary band. This population of neurons has been described as dopaminergic (6). At 72 hpf the hybridization signal associated with *Sp-SecV* and *Sp-AN* NP expression is specifically revealed in two groups of cells bilaterally arranged in a manner that resembles the lateral ganglia neurons (Figures 4A,B). The expression of *Sp-SecV* and *Sp-AN* NP gene suggests a neurosecretory role of these cells. Finally, a probe against the *Sp-Nesf* NP gene shows a diffuse signal in the ciliary band, not restricted to any particular subpopulation of cells (Supplementary Figure 6E).

Taken together our data revealed that several NP genes are expressed in single or groups of cells and identifies several neuronal and neuronal-like sub-types in both the central and peripheral nervous system of sea urchin larvae. Each subtype of cells is characterized by a specific combination of expressed NP genes.

#### Mouth and gut cell systems

**Figure 5:**
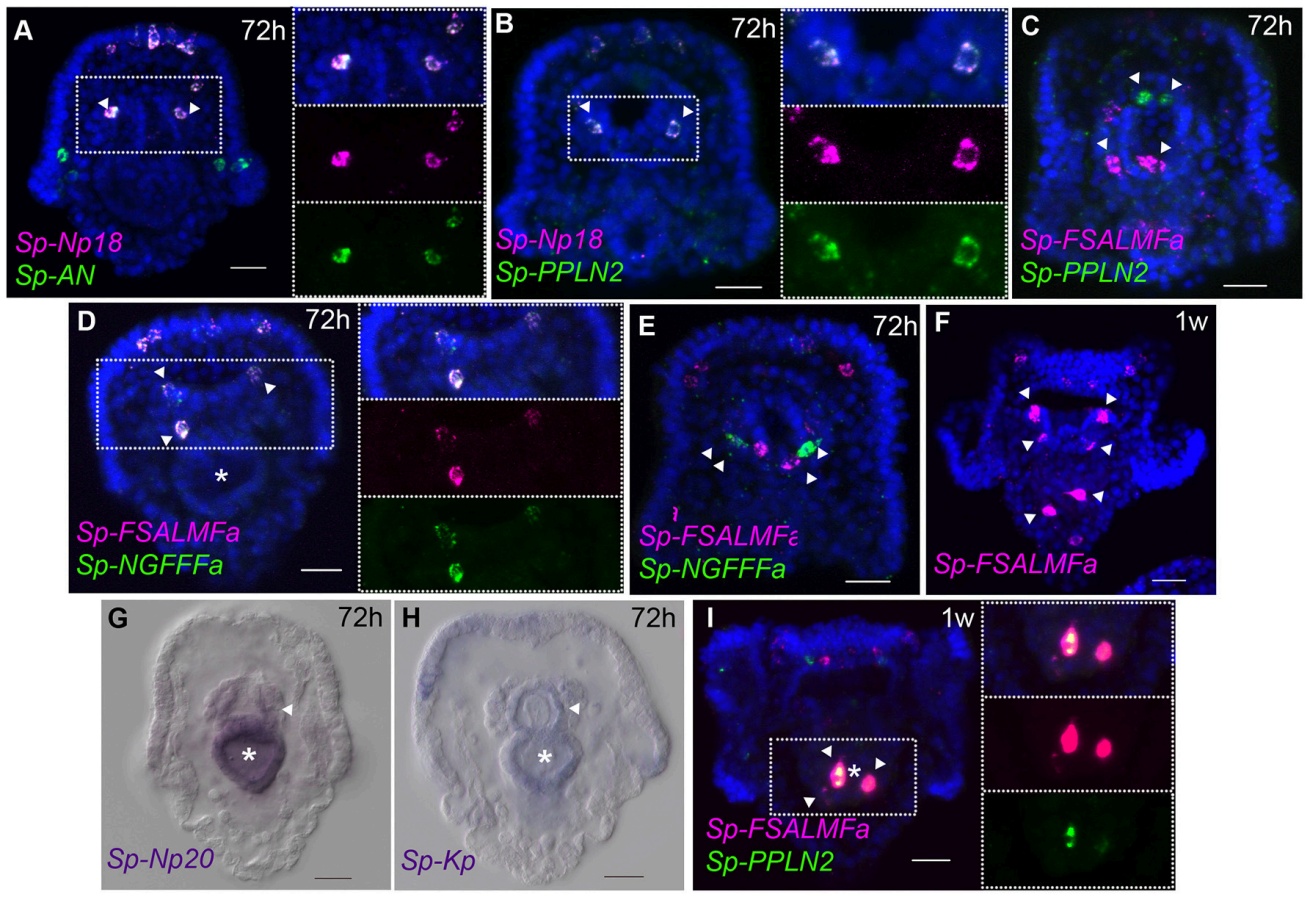
Expression of NP genes in the mouth and gut cell systems. **(A–F,I)** Merged and single channel confocal images of pluteus larvae labeled using single and double fluorescent ISH. **(G,H)** Chromogenic whole-mount ISH of *Sp-Np20* and *Sp-Kp* NP gene expression in pluteus larvae. Bottom-left corner indicates the probe used. Top-right corner indicates the larval stage in hours or weeks post-fertilization. Dotted white boxes highlight the magnified region shown to the right. Asterisk indicates the midgut. Scale bars: 20 μm.

Several neurons expressing GABA or dopamine have been described in the mouth and in the esophagus of the sea urchin larva (6). At the early pluteus stage (72 hpf), approximately four to six neurons appear in the lip and these have been described as dopaminergic neurons (6), while a few neurons of endodermal origin differentiate in the esophagus (10). Single and double whole-mount ISH experiments show that *Sp-FSALMFa, Sp-NGFFFa, Sp-Np18, Sp-PPLN2*, and *Sp-AN* NP genes are all expressed in two to four isolated and bilaterally arranged cells around the mouth (Figures 5A–F) in the 72 hpf larvae. Although there is variation in the number of cells and the degree of co-expression of NP genes around the mouth, signals associated with *Sp-PPLN2, Sp-AN*, and *Sp-Np18* expression are largely co-localized in two to four cells in the larval lip, as they are co-localized in the apical domain (Figures 3D,G, 5A,B). The *Sp-FSALMFa* and *Sp-NGFFFa* NP genes are mostly co-expressed in another population of cells around the mouth of the larva (Figure 5D), which rarely coincide with the cells expressing *Sp-PPLN2* (Figure 5C). Even the cells expressing the *Sp-FSALMFa* and *Sp-NGFFFa* NP genes around the mouth show a high degree of variability of co-expression (compare Figure 5D with Figure 5E and Supplementary Table 4). The number and type of neurons around the mouth increase in the larva, as well as the number of NP gene-expressing cells, as shown by the expression of *Sp-FSALMFa* (Figure 5F) in 1 week old larvae, which is consistent with the EdU staining shown in Figure 3A. To better understand the variation in NP genes expressed in various cells we analyzed several (*N* > 7, with exception of *Sp-FSALMFa* and *Sp-Np18* NP genes where *N* > 2) larvae in each experiment. Supplementary Table 4 summarizes the results and shows that the single cells surrounding the mouth never express *Sp-Trh* or *Sp-SecV* (Figures 3C,H)*. Four* double fluorescent ISH experiments are analyzed in the 72 hpf and 1 week old larval mouth. The co-expression of *Sp-Np18* with either *Sp-AN* or *Sp-PPLN2* shows a high degree of variability when several embryos are analyzed. In contrast, *Sp-FSALMFa* with *Sp-NGFFFa* fluorescent ISH experiments generally show a more consistent co-expression in the cells around the mouth (Supplementary Table 4).

Two to three GABAnergic neurons have been identified in the early pluteus foregut (6). Chromogenic ISH hybridization reveals the expression of two NP genes, *Sp-Np20*, and *Sp-Kp*, with a diffuse pattern in the foregut and not only in individual neurons (Figures 5G,H). Indeed, these two NP genes are also expressed in the entire midgut. Later in larval development, at 1 week-old, *Sp-FSALMFa, Sp-NGFFFa* (Supplementary Figures 6F,G) and *Sp-PPLN2* are co-expressed in two to three isolated cells in the mid-gut (Figures 5F,I). There is also variability in the expression of *Sp-FSALMFa* and *Sp-NGFFFa*—compare Supplementary Figure 6F with Supplementary Figure 6G. None of the other NP genes analyzed in this study were detected in these cells, not even in 1-week old larvae (Figure 5I; Supplementary Figure 6C). Neuronal cell types have so far not been described to be in association with the midgut, however other cell types with endocrine/digestive function are known to be present in the midgut (45). Our results reveal a similar situation as described for the central and peripheral nervous systems: several cell populations are identified by specific NP gene expression signatures. Furthermore, our fluorescent ISH analysis has revealed a variable pattern of NP gene expression in the neuron-like cells around the mouth and the presence of a specific population of cells in the midgut.

## Discussion

**Figure 6:**
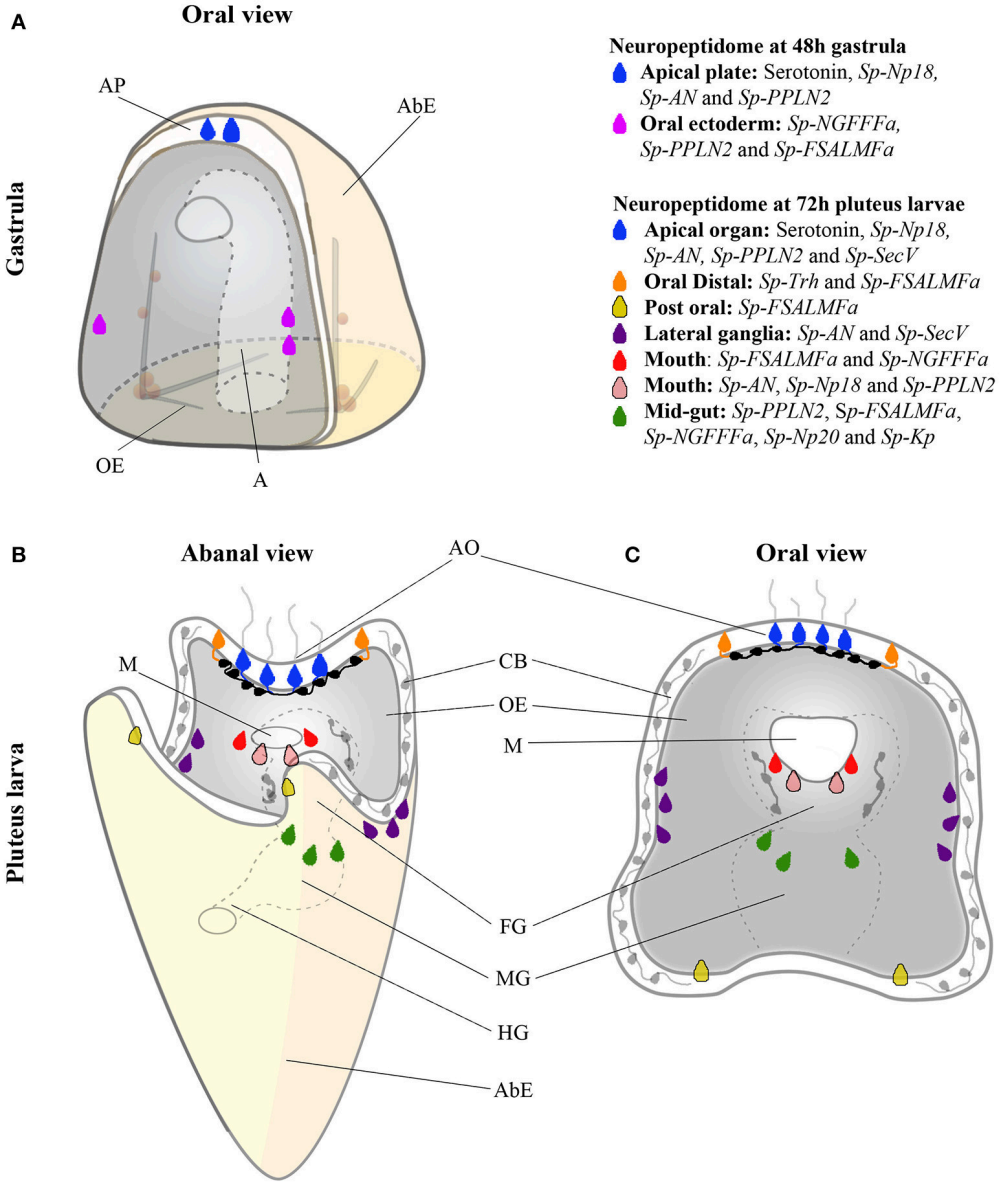
Color coded cartoon map showing various combinations of NP gene and *Sp-SecV* expression at single cell resolution in the sea urchin at the gastrula stage and in pluteus larvae. **(A)** NP gene expression in two populations of cells at the gastrula stage. Serotonergic neuronal precursor cells (blue) and post-oral neuronal-like precursor cells in the oral ectoderm (magenta). **(B,C)** NP gene expression in eight different neuronal and presumed neuronal populations in the larval nervous system, shown from abanal and an oral view. Sensory serotonergic neurons (blue), lateral ganglion neurons (purple), presumed oral distal neurons (orange), presumed post-oral neurons (yellow), presumed lip neurons (red and pink), and mid-gut exocrine-like cells (dark green). Black and gray neurons (in foregut, ciliary band, and serotonergic interneurons) have been identified in previous studies but do not express any of the NP genes examined in this study. Abbreviated labels refers to the following, AP (Apical Plate), AbE (Aboral Ectoderm), OE (Oral Ectoderm), A (Archenteron), AO (Apical Organ), CB (Ciliary band), M (Mouth), FG (Foregut), MG (Midgut) and HG (Hindgut).

The early sea urchin larval nervous system is composed of 40–50 neurons, characterized by the expression of specific neurotransmitters (5, 6). Understanding the diversity and distribution of NP gene expression is essential for identification of the diverse neuronal subtypes in echinoderm larval nervous systems. Here we report the first multi-gene analysis of NP gene expression in larvae of a sea urchin species (Figure 6). *S. purpuratus* has at least 38 NP genes and expression of almost all NP genes analyzed here was detected in the developing sea urchin or larval stages. Spatial expression patterns of nine NP genes were revealed in the early pluteus larvae and the expression of five NP genes was detected already in the gastrula. The existence of diverse neuronal subtypes in the echinoderm larval nervous system has been revealed by immunocytochemical analysis of the neurotransmitters serotonin, dopamine and GABA (6). Localization of NP gene expression, as reported here, has revealed distinct sub-populations of neuropeptidergic cells, demonstrating that the sea urchin larval nervous system is far more complex than previously thought (Figure 6). Furthermore, QPCR analysis detected the expression of a few NP genes prior to the gastrula stage, suggesting that these signaling molecules also have a role during the early development of the sea urchin embryos. Our spatial analysis shows no evidence of localization to known neuronal precursor cells (data not shown) and therefore NPs may be involved in signaling associated with non-neural developmental processes.

Neuronal co-expression of multiple neuropeptide precursor genes and/or neuropeptides derived from different precursor proteins has been reported in a variety of taxa including, for example, cnidarians (46), molluscs (47), annelids (48), and vertebrates (49). Here we report this property of neuropeptide signaling systems for the first time in the larval nervous system of an echinoderm. Thus, our results show that consistent co-expression signatures were found for two different combinations of NP genes: (i) *Sp-FSALMFa* and *Sp-NGFFFa* and (ii) *Sp-AN, Sp-PPLN2*, and *Sp-Np18*. The former two genes are initially co-expressed in cells that resemble the precursor of postoral neurons in the gastrula and then in neuron-like cells around the mouth, while the latter three genes are expressed in the primary sensory neurons located in the apical organ. Their co-expression starts as soon as the precursor sensory cells express the serotonin marker at the gastrula stage (48 hpf) and then remains in these cells in all subsequent stages analyzed here. The same three genes are also co-expressed in neuron-like cells around the mouth at the pluteus stage (Supplementary Table 4). These co-expression signatures suggest similar regulation of these genes, possibly by a combination of transcription factors, which may work as terminal selector genes to control the differentiated state of post-mitotic neurons (50). Recent studies have shown the role of several transcription factors and signaling molecules in determining the identity of large sub-populations of neurons [for review see (4)], including, for example, *Ac-sc* regulation of the development of serotonergic neurons (51). However, none of the data so far published can explain the highly restricted expression pattern of NP genes that we have identified in this study. This suggests that more studies need to be done to investigate the regulation of neuronal subtypes. Accordingly, a paper by Perillo and collaborators in this issue (52), identifies for the first time a precise combination of transcription factors (*Lox, Brn1/2/4*, and *Islet-1*) that is consistent with the restricted expression of the *Sp-AN* NP gene in the lateral ganglia neurons.

A few distinct sub-populations of neurons and neuron-like cells have been identified in this study, which have not been described before. For instance, the isolated cells associated with the ciliary bands, at the base of the arms, the oral distal and the post-oral, all express *Sp-FSALMFa* while the oral distal cells also express *Sp-Trh*. Perillo et al. (52) also identified *Sp-AN* NP gene expression in the post-oral neurons. Two types of neurons have been recently described in the ciliary bands of sea urchin larvae: cholinergic neurons are widely distributed throughout this structure and dopaminergic neurons are concentrated in a post-oral location (51). Adams and collaborators identified the post-oral dopaminergic neurons to be involved in controlling arm length in response to food (53). *Sp-FSALMFa* is expressed in four cells, one at the base of each arm and so neuropeptides encoded by the *Sp-FSALMFa* gene may likewise have a role in controlling arm length or arm growth, possibly in response to an environmental signal. In comparison, the *Sp-Trh* NP gene is expressed only in two cells and these are at the base of the oral distal arms. Interestingly, TRH is known to have a role in growth in vertebrates and in the nematode *C. elegans* (54–56) and therefore it is plausible to hypothesize that TRH-type peptides may also affect arm growth in *S. purpuratus* larvae, perhaps asymmetrically. Furthermore, *Sp-Opsin3.2* has been detected in two cells, adjacent to the apical plate (57), in a similar position to the *Sp-Trh* positive cells. Double fluorescent ISH will reveal if these two genes are co-expressed, suggesting a light-sensing role of the cells expressing the *Sp-Trh* NP gene. Taken together, these observations suggest that neuropeptides expressed in the cells at the base of the arms may be involved in controlling arm length in response to food and/or light.

Neuropeptides derived from precursor proteins are packed in dense core vesicles (DCV). The maturation and release of functional neuropeptides is regulated by several factors, among them the family of chromogranin/secretogranin proteins, which play a key role in the regulated secretory pathway (58). In this study we show the expression of *Sp-SecV*, previously identified in proteomic studies (25), specifically in the primary sensory neurons of the apical organ and in the pancreatic-like neurons of the lateral ganglia (52). Interestingly, human Secretogranin V (SGC5, Gene ID 6447) is specifically expressed in the brain, pancreas, adrenal and stomach, highlighting the conservation in deuterostomes of an ancient neuroendocrine molecular pathway.

Our quantitative analysis of the whole-mount ISH results (Supplementary Tables 4, 5) reveals variability of the NP gene combinations expressed during development and larval stages, suggesting a constant modulation of the expression of NP genes, in response to both developmental and possibly environmental cues. For instance, the post-oral neuron precursors seem to exhibit a transient expression of NP genes between 48 and 72 hpf: at 48 hpf, they specifically express *Sp-FSALMFa, Sp-NGFFFa* and *Sp-PPLN2*, whereas only the *Sp-FSALMFa* precursor gene is expressed at 72hpf. A similar combination of NP genes is expressed in the lip and midgut cells at 72 hpf, suggesting that these newly formed neuronal or secretory cells might acquire a similar NP gene signature. Therefore, the two initial post-oral neuron-like cells (Figure 6) either turn off *Sp-NGFFFa* and *Sp-PPLN2* NP genes, or the cells at 72 hpf are actually newly differentiated, consistent with the constant production of neurons identified by EdU labeling (Figure 3A) (41). Cell-tracing experiments have not been performed on those cells, and so despite previous literature referring to both sets of neurons as “post-oral” we cannot be sure these are the same cells. In both situations, our analysis identifies a highly dynamic regulation of NP genes in the larvae, as already discussed for neuron-like cells associated with the mouth (Supplementary Table 3).

Figure 6 summarizes the expression of NP genes at the gastrula (48 hpf) and pluteus (72 hpf) stages. At the gastrula stage the first NPs genes are expressed in a pattern consistent with the appearance of post-mitotic precursors of serotonergic neurons and post-oral neurons (41). Furthermore, two distinct combinations of NP genes are expressed in each population of cells consistent with these neuronal precursors, overlapping only by the expression of *Sp-PPLN2*, suggesting they have diverse functions. In the early pluteus (72 hpf) the number of NP genes expressed (Figure 1) largely correlates with the increased complexity of the nervous and secretory systems in the apical plate, ciliary band, lateral ganglia, lip, and foregut. At the early pluteus stage, the NP genes are expressed in a pattern suggesting the apical plate and lip each have at least two distinct sub-populations of neurons and neuron-like cells. The expression of *Sp-FSALMFa* in the post-oral dopaminergic neuron-like cells and *Sp-FSALMFa* and *Sp-Trh* in the oral distal neuron-like cells identifies two distinct populations of cells, separate from rest of the ciliary band. Taken together, we propose the following populations of neuronal and/or secretory cells in the sea urchin pluteus (Figure 6): the apical plate is divided into: (1) primary sensory serotonergic neurons that express various NP genes and *Sp-SecV*; and (2) serotonergic interneurons that do not express any of the NP genes investigated; (3) the oral distal neuron-like cells in the ciliary band, adjacent to the apical plate, characterized by the expression of *Sp-Trh* and *Sp-FSALMFa* NP genes; (4) two post-oral neuron-like cells associated with the ciliary band, at the bases of the post-oral arms, identified by the expression of *Sp-FSALMFa*; (5) other ciliary band neurons generally identified by SynB neuronal markers (5) and expressing *Sp-Nesf* ; (6) lateral ganglia neurons associated with the left and right ciliary bands and expressing *Sp-AN* (52) and *Sp-SecV* NP genes; (7) the larval neuron-like cells associated with the mouth, which are divided into at least two sub-types, each with a distinct combination of NP gene expression; (8) foregut GABAergic neurons described by Bisgrove and Burke (6) not expressing any of the NP genes studied here; and (9) midgut cells expressing NP genes.

In conclusion, our study has revealed that the sea urchin larval nervous system is far more complex at a neurochemical level than was previously known. Our NP gene expression map provides the basis for future work, aimed at understanding the regulation and function of diverse neuropeptides in the sea urchin larval nervous system.

## Author contributions

PO and MRE originally conceived the study. NJW, TM, LW, MLR and MP contributed to the acquisition of data. NJW, TM, MIA, MRE and PO contributed to the interpretation of the data. NJW and PO wrote the first draft of the manuscript. All authors contributed to manuscript revision, read and approved the submitted version.

### Conflict of interest statement

The authors declare that the research was conducted in the absence of any commercial or financial relationships that could be construed as a potential conflict of interest.
