# Supplementary material for: Neuropeptidergic Systems in Pluteus Larvae of the Sea Urchin Strongylocentrotus purpuratus: Neurochemical Complexity in a “Simple” Nervous System
Source: Front Endocrinol (Lausanne). 2018 Oct 25;9:628. doi: 10.3389/fendo.2018.00628 (PMC6209648; doi:10.3389/fendo.2018.00628)
Supplement: Supplementary file 1 [file Presentation_1.pdf]

Supplementary Material

Neuropeptidergic Systems in Pluteus Larvae of the Sea Urchin *Strongylocentrotus purpuratus*: Neurochemical Complexity in a "Simple" Nervous System

Natalie J. Wood[1†], Teresa Mattiello[1,2†], Matthew L. Rowe[1,3], Elizabeth Ward[1,4], Margherita Perillo[2], M. Ina Arnone[2], Maurice R. Elphick[3]* and Paola Oliveri[1]*

*** Correspondence:** Corresponding Authors: p.oliveri@ucl.ac.uk, m.r.elphick@qmul.ac.uk

1 Supplementary Figures, Tables and Extended Methods

Supplementary Table 1. Clone and probe information for nine NP genes, *Sp-SecV and Sp-SynB*.

NP gene	RNSP-clone	Length of probe (nucleotides)	Polymerase used to make antisense	Forward primer	Reverse primer
Sp-FSALMFa	5H7	2000	Sp6	Radial Nerve cDNA Library	Radial Nerve cDNA Library
Sp-NGFFFa	5L15	1650-2000	Sp6	Radial Nerve cDNA Library	Radial Nerve cDNA Library
Sp-Trh	9P21	3900	Sp6	Radial Nerve cDNA Library	Radial Nerve cDNA Library
Sp-AN	5K1	2155	Sp6	Radial Nerve cDNA Library	Radial Nerve cDNA Library
Sp-Np18	9L6	1650	Sp6	Radial Nerve cDNA Library	Radial Nerve cDNA Library
Sp-PPLN2	5B10	3700	Sp6	Radial Nerve cDNA Library	Radial Nerve cDNA Library
Sp-SecV	9I5	900	T7	Radial Nerve cDNA Library	Radial Nerve cDNA Library
Sp-Np20	n/a	1323	Sp6	TGTCTAAGCCTCCAATCCGG	CGAATAAGGTGCCACTCCCT
Sp-Kp	n/a	1410	Sp6	CCACCGCACTATCATTTGACC	CCTTCTCCCATCCCATTTGTAAT
Sp-SynB	n/a	1017	T7	GAACAAGATAAGCGACGTGATG	TGTCAACCAACCTGATAGTCCA
Sp-Nesf	n/a	955	Sp6	TAGTGCATGTAGCCCAGCAG	GAAGCAGCCTCCATGAACTC

Supplementary Table 2. *Sp-SecV* and NP gene names, abbreviated names, identification and QPCR primer sequences.

			**QPCR primer sequences**	
**NP gene name**	**Abbreviated NP gene name**	**Gene ID. (Echinobase ID. or NCBI ID.)**	**Forward (5'-3')**	**Reverse (5'-3')**
Sp-Neuropeptide precursor 13	***Sp-Np13***	XM_001176371.3	GTCACTTCCGGCCAATCTTG	GTCTAATGTGGTGGGTGGCT
Sp-Neuropeptide precursor 8	***Sp-Np8***	XR_143667.2	CCCTCGCCTTTATCGTCTCT	GTGCCTTTATCGGGTAGTGC
Sp- Neuropeptide precursor 9	***Sp-Np9***	XR_143632.2	TTTAGCCGTTCTCCTCCTCG	CCTTGCATTGTTTCCGCTGT
Sp- Gonadotropin-releasing hormone 2	***Sp-GnRH2***	XR_971124.1	CGGCTCAGCGGTAGATACT	TGTGGAAGGTCAGGTCGTAC
Sp-Melanin-concentrating hormone	***Sp-MCH***	GI: 115958765	GTCACATGATCGACGGTTTCA	TCTACCCGATCTGCTCCTCT
Sp-Eclosion hormone 2	***Sp-EH2***	XR_972749.1	GATGACGTCTTCTCCCGATT	GCAGTTTCCGAGCTTGTAGG
Sp-Somatostatin 2	***Sp-SS2***	XM_001176809.3	GCCTGTCATGGAACTGCAAG	GTCTACGGCTTGGTCCATCT
Sp-Somatostatin 1	***Sp-SS1***	XP_001176669.1	GGGAACTTGGAGACGCAGAT	TCGAATCCTCTTGAGGTGGC
Sp-Pigment-dispersing factor	***Sp-PDF***	XP_001192435.1	ACGTGGGATGATGCAGAAGA	CTTGAGCGCTTTCCTGCTTT
Sp-Orexin1	***Sp-Ox1***	XM_011677444.1	GACAGACACAGCAGAAACCG	TATGGTGATGGCCCTGTGTT
Sp-Orexin2	***Sp-Ox2***	XR_973664.1	TGCAACCTCAGAAGCGATTG	AGTGATGCCAACTCCGCTAT
Sp-Neuropeptide precursor 17	***Sp-Np17***	XM_001177757.3	CGATATGTGTCCACCAAGCC	TTCAATACGCCTGTCCTCCA
Sp-Echinotocin	***Sp-Echino***	SPU_006899	AACTCCCAGGAAACCCTTGT	TAACCCGGAGGTCTTTCCTC

Sp-Ftype SALMFamide	***Sp-FSALMFa***	SPU_021555	AAACGTACGACTGGGTCCAC	CATCTCTGCGTTTCGTTGAA
Sp-Ltype SALMFamide	***Sp-LSALMFa***	XR_973850.1	AGTTGCATATGCCCAAGAGG	GAATGCTGCCCATGTTCTTT
Sp-NGFFFamide	***Sp-NGFFFa***	SPU_030074	CCATCATCACGAAGCAGAGA	TCCCTGGGTGAGTTTACAGC
Sp-Gonadotropin-releasing hormone	***Sp-GnRH***	SPU_019680	CGCAGAAGTCAACTCGAACA	ATTCGATGTCGCATCATTCA
Sp-Thyrotropin-releasing hormone	***Sp-Trh***	SPU_008352	GCCAGTACCCAGGTGGTAAA	CGTAGCTCAGGCGATGGTAT
Sp-AN	***n/a***	SPU_018666	GTGACGATTTCGGTGATGAA	TCCTCTGAAGTAGTTCGCTCTC
Sp-Calcitonin	***Sp-Calc***	XR_972762.1	CCAACAGAGACGGACTCTCA	CACTCGGTTCTTTGCCACTT
Sp- Neuropeptide precursor 10	***Sp-Np10***	XR_971715.1	GGAGAGGTGCAGCTGAGAAC	TTGCCGCTGTTCAGAAGATT
Sp-Adam/Tsl6	n/a	SPU_003170.4a	ACGTAAACGCCCTCACATTC	CTTGTGGGCTTCTGATCTCC
Sp- Neuropeptide precursor 18	***Sp-Np18***	XM_001175944.3	CACAAGCCGTTTGCAGTCTA	AAGCACTTCTTTGCGCAGTT
Sp- Neuropeptide precursor 11	***Sp-Np11***	XR_973214.1	ACGAAGATGCAATGGACCTC	ATACACTCCGGCATCGTCAT
Sp-Cholecystokinin	***Sp-CCK***	WHL22.619425.0	GAACTATGACCCGCAACCAT	GCCGAGTTCACCGTAGAGTC
Sp-Pedal peptide-like neuropeptide 2	***Sp-PPLN2***	SPU_024381	TGGACACTACCGATTGAGGA	TTGCATCGGTTCCATGTTTA
Sp-Glycoprotein hormone 3	***Sp-GPH3***	SPU_011451	CAGGTCCCATCGCTTCTTAC	AAGTGAAGGTCGGGTCAATG
Sp-Kisspeptin	***Sp-Kp***	WHL22.176298.0	http://www.echinobase.org/Echinobase/	http://www.echinobase.org/Echinobase/
Sp- Neuropeptide precursor 20	***Sp-Np20***	SPU_014142	http://www.echinobase.org/Echinobase/	http://www.echinobase.org/Echinobase/

Sp-Nesfatin	***Sp-Nesf***	WHL22.529220	http://www.echinobase.org/Echinobase/	http://www.echinobase.org/Echinobase/
Sp-SecretograninV	***Sp-SecV***	SPU_015798	AACCCAATCCCTGAGGTTTC	TCACATGCACACACCTGATG
Sp-Glycoprotein hormone 1/2	***Sp-GPH1/2***	SPU_004405 / SPU_005842	AAGTCTTCGCACCACGAGAT	GTTTCGGCATTTGCAACTCT
Sp-Buriscon alpha-like	***Sp-Bursα***	SPU_003984	GTTGATGTTGGCCCTGATTC	CCTACCGAGTCCAAGGTGAC
Sp-Buriscon beta-like	***Sp-Bursβ***	SPU_017707	AACCAATGCGAGGGTAAATG	TGTGATTCCTAGGGCGTGAT
Sp-Ubquitin	***Sp-Ubq***	SPU_021496	CACAGGCAAGACCATCACAC	GAGAGAGTGCGACCATCCTC
Sp-18S-rRNA	***Sp-18S***	L28055	CAGGGTTCGATTCCGTAGAG	CCTCCAGTGGATCCTCGTTA

Supplementary Table 3. Embryonic expression of NP genes across developmental time from 24hpf to 70hpf as determined by Quantitative PCR (QPCR). Data are indicated as number of transcripts per embryo.

	**Developmental time (hpf)**								
**NP gene**	**24**	**27**	**30**	**33**	**40**	**45**	**48**	**52**	**70**
Sp-Np13	34	38	11		14	31	45	137	222
Sp-Np8	68	78	80		180	240	123	142	195
Sp-Np9	70	62	34		88	204	284	641	2690
Sp-GnRH2	202	212	12		9	19	49	121	957
Sp-MCH	161	154	19		7	15	33	186	356
Sp-EH2	6	17	1		11	7	8	21	73
Sp-SS2	16	47	5		18	6	14	53	1272
Sp-SS1	321	279	141	140	139	77	68	88	134
Sp-PDF	8	8	12	1	20	37	56	65	623
Sp-Ox1	359	316	575	241	241	689	1255	2610	6405
Sp-Ox2	288	477	343	75	40	46	52	107	517
Sp-Np17	19	94	14	31	14	22	31	87	209
Sp-Echino	68		34		96		200		64
Sp-FSALMFa	64		128		164		346		2167
Sp-LSALMFa	7		5		0		11		257
Sp-NGFFFa	34		11		33		19		108
Sp-GnRH	0		0		0		0		0

Sp-Trh	618		250		210		82		426
Sp-AN	26		7		120		655		9377
Sp-Calc	28		8		31		6		39
Sp-Np10	25		39		114		63		71
Sp-Adam/Tsl6	4		44		32		73		11
Sp-Np18	220		196		317		485		4943
Sp-Np11	1		1		27		23		122
Sp-CCK	105		156		601		391		1695
Sp-PPLN2	62		79		430		830		451
Sp-GPH3	9		19		432		1476		3262
Sp-SecV					1664	6497	13471		70876
Sp-GPH1/2					1415	3717	10450		42529
Sp-Bursα					813	1958	5652		9011
Sp-Bursβ					155	464	853		3750

Supplementary Figure 1. A graph representing the reproducibility of four technical replicas of embryonic QPCR data. Developmental times from 24 hpf to 70hpf are plotted on the X-axis; average of cycles at the threshold (Ct) values are plotted on the Y-axis. Error bars represent the standard deviation of the four technical replicas. Genes with a standard deviation less than 5 are in blue and genes with a standard deviation greater than 5 are in red. The lower the Ct value, the higher the initial number of transcripts present in the cDNA. Generally, a lower level of expression generates high Ct values with a higher standard deviation. The *Sp-GnRH* NP gene is not shown because no Ct value is identified after 40 cycles of amplification in any of the developmental stages and therefore we conclude that it is not expressed (see Figure 2 for larval expression of *Sp-GnRH*, which serves as a positive control for the *Sp-GnRH* QPCR primers).

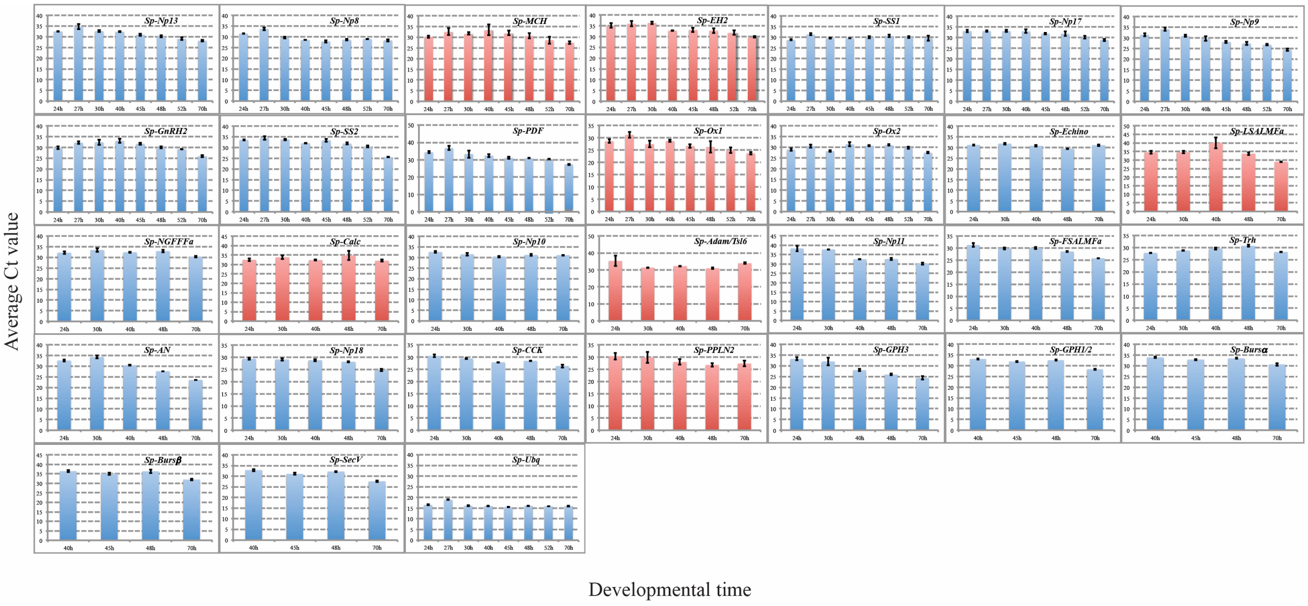

Figure 2: A graph representing the reproducibility of four technical replicas of larval and juvenile QPCR data. Stages 70hpf, 5 weeks and juvenile are plotted on the X-axis; average of cycles at the threshold (Ct) values are plotted on the Y-axis. Error bars represent the standard deviation of the four technical replicas. Genes with a standard deviation less than 5 are in blue and genes with a standard deviation greater than 5 are in red. The lower the Ct value, the higher the initial number of transcripts present in the cDNA. Generally, a lower level of expression generates high Ct values with a higher standard deviation.

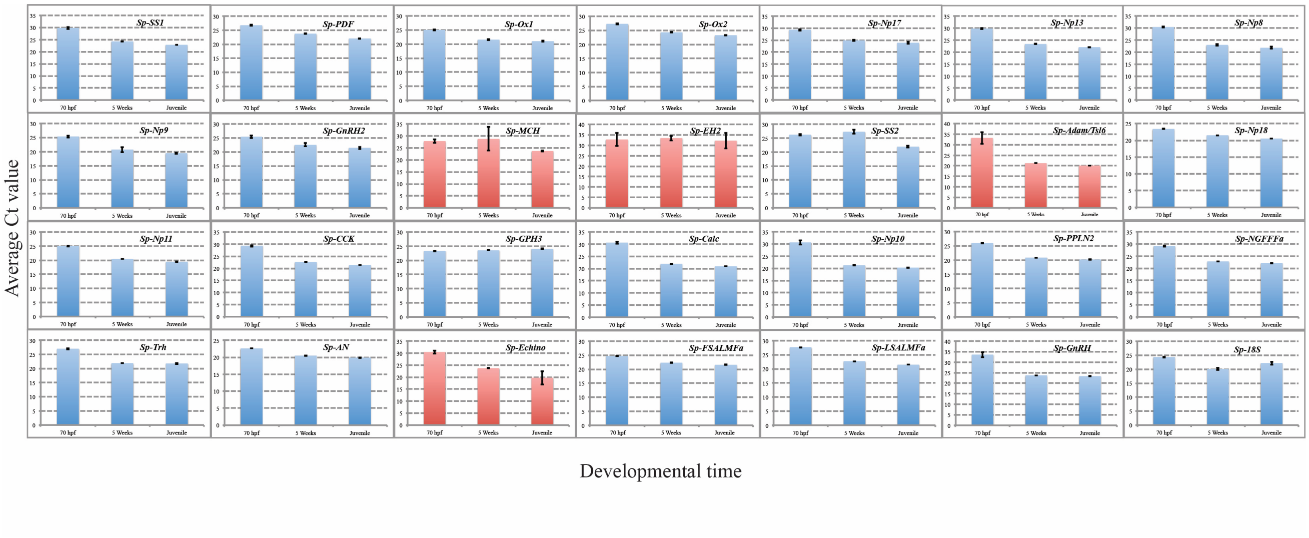

Supplementary Figure 3. The relative expression of three NP genes using a publically available transcriptome data set (Echinobase: http://www.echinobase.org/Echinobase/). The relative expression (individual maximum expression) is shown for *Sp-Kp* (blue), *Sp-Nesfatin* (red) and *Sp-Np20* (green) NP genes from 24hpf to 72hpf.

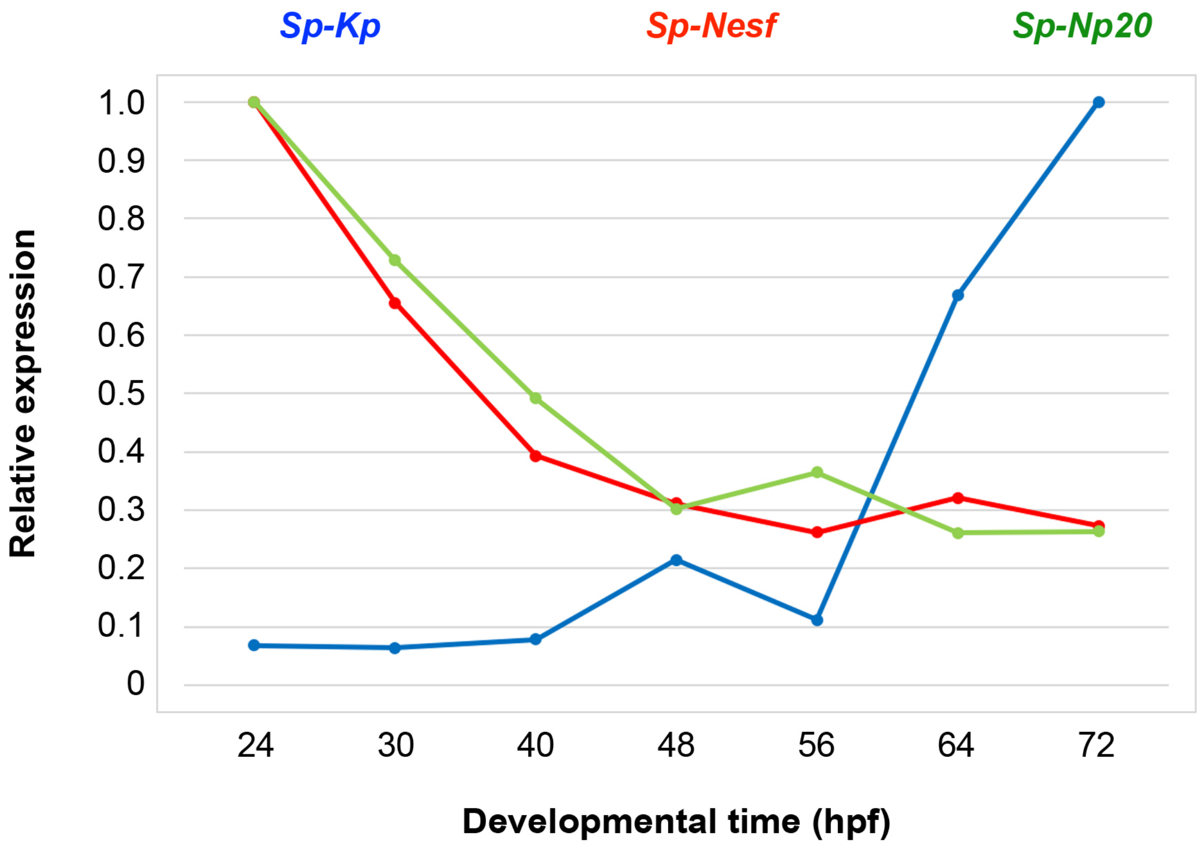

Supplementary Figure 4. A comparison of the relative expression of nine NP genes determined by QPCR (this paper) and by transcriptome sequencing (URL:http://www.echinobase.org/Echinobase/). (A) The relative expression (individual maximum expression) of five NP genes, for which we show fluorescent *in situ* hybridization (FISH) data, *Sp-FSALMFa* (blue), *Sp-NGFFFa* (red), *Sp-Trh* (green), *Sp-PPLN2* (orange) and *Sp-AN* (purple) are mostly comparable in QPCR and transcriptome data. (B) The relative expression (individual maximum expression) of four NP genes, *Sp-SecV* (blue), *Sp-GPH1/2* (red), *Sp-Bursα* (green) and *Sp-Bursβ* (orange) are mostly comparable in QPCR and transcriptome data and show a general increase in relative expression in both QPCR and transcriptome data between 48hpf and 72hpf.

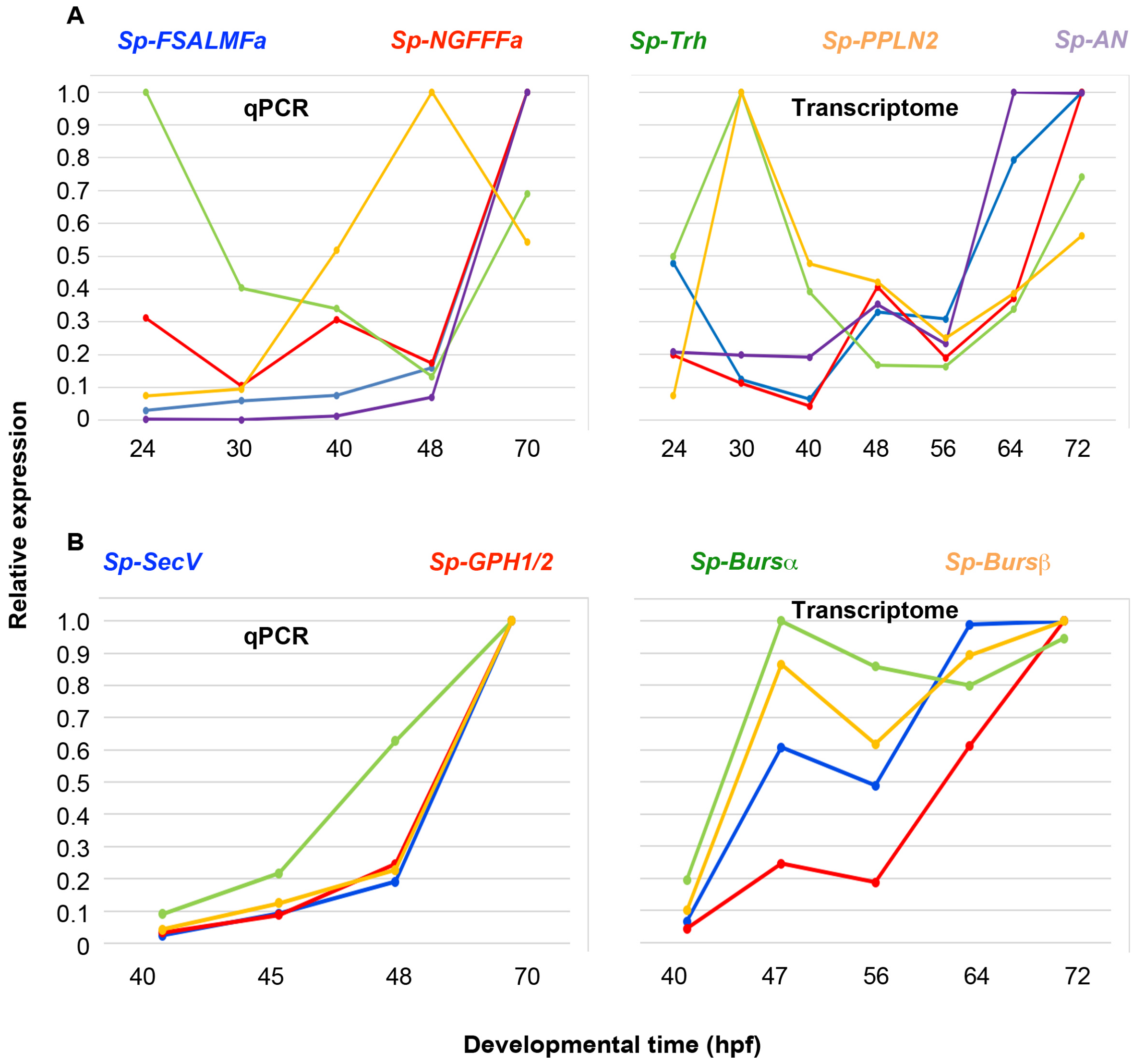

Supplementary Figure 5. Relative expression of 27 NP genes during larval and juvenile development. The relative expression (individual maximum expression) of these NP genes are divided into line graphs A-D based on their expression profile. (A) Ten NP genes have a peak in expression at the juvenile stage. (B) Nine NP genes have a peak in expression at the juvenile stage, but preceded by a decrease in expression at 5 weeks of larval development. (C) Six NP genes have no or little expression in larval stages and a considerable increase in juveniles. (D) Two NP genes are expressed in the early larva (70 hpf) but then exhibit reduced expression in late larvae and juveniles.

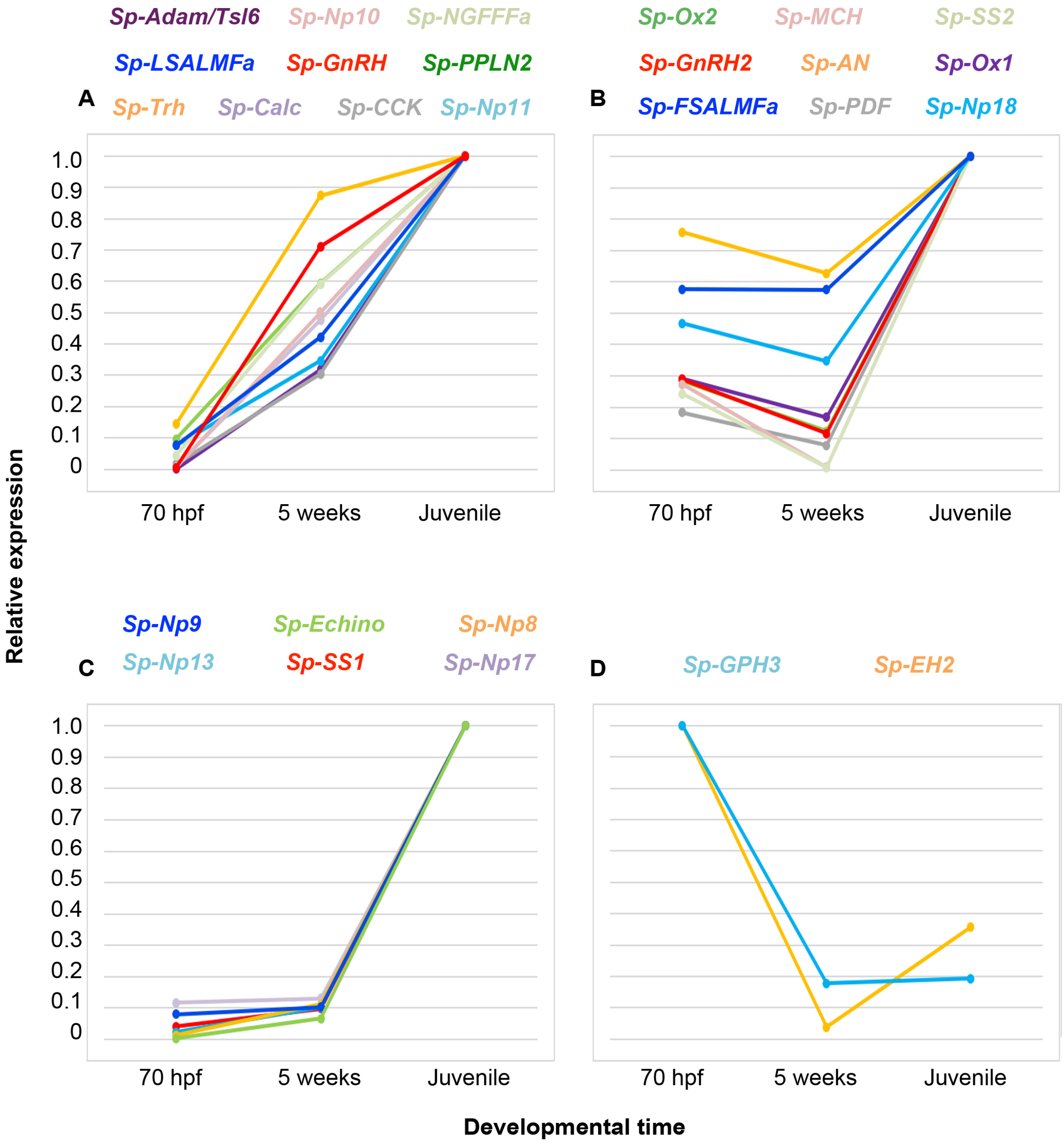

Supplementary Figure 6. Expression of NP genes in the ciliary band, gut and apical plate and EdU staining. Maximum projection of merged confocal images of single and double FISH, and immunohistochemistry. (A) *Sp-Np18* NP gene and *Sp-SynB* probe co-expressed in serotonergic sensory neuronal precursor cells in gastrula embryo. (B) EdU labeled larva stained with SynB antibody (1E11) that reveals neuronal cells (Nakajima *et al.*, 2004). Dividing cells labeled with EdU are mostly located in the neurogenic tissues. Generally the $SynB^{+}$ neurons are not dividing (yellow arrowhead), although some EdU^{+} cells are also $SynB^{+}$ when analyzed in single confocal z-slices (white arrowhead). (C) *Sp-Trh* NP expressed in cells at the base of oral distal arms, connected to serotonergic ganglion by long projection in a pluteus larvae. (D) *Sp-FSALMFa* NP gene expressed in presumed oral distal and post oral neurons of a pluteus larva. White arrows indicate presumed oral distal neurons and yellow arrows indicate presumed post oral neurons. (E) *Sp-Nesf* NP gene expression in the ciliary band of a pluteus larva, showing diffuse ciliary band staining. Cilia labeled by acetylated tubulin. (F and G) *Sp-FSALMFa* and *Sp-NGFFFa* NP genes both co-expressed and differentially expressed in the mid-gut of a pluteus larva. (H) *Sp-PPLN2* and *Sp-Np18* co-expressed in sensory serotoninergic neurons in the apical plate of 1 week old larva. Bottom-left corner indicates the probe or antibody used. Top-right corner indicates the larval stage in hours' (h) or weeks' (w) post fertilization. Dotted white boxes highlight the magnified region shown to the right. Scale bars: 20μM.

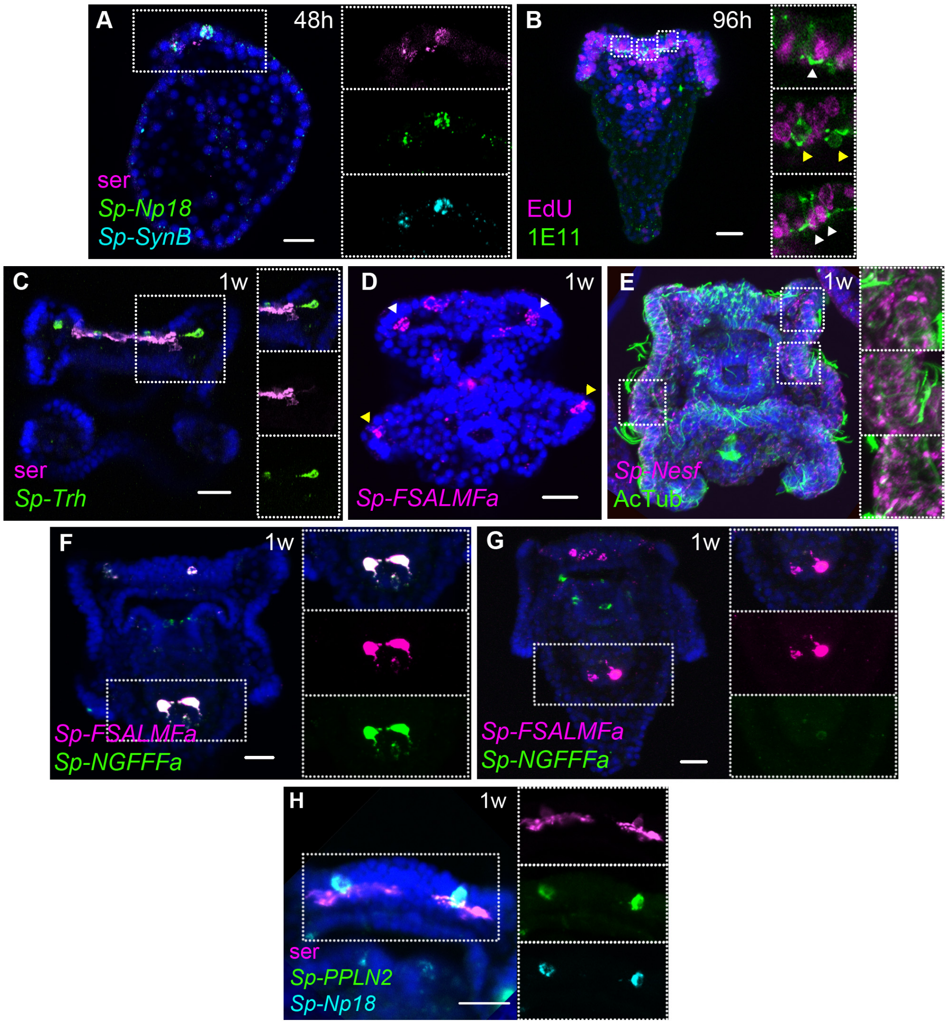

Supplementary Table 4. Four pairs of NP genes exhibit variable gene expression in cells around the mouth at larval stages (72hpf and 1 wpf). The number of embryos with a particular gene expression pattern around the mouth with respect to the total number of embryos imaged by double fluorescent WMISH is shown. Generally, these four pairs of NP genes have variable expression patterns between individual embryos imaged. *Sp-AN* and *Sp-NP18*, and *Sp-PPLN2* and *Sp-Np18 NP* gene pairs have a more variable expression pattern. *Sp-FALMFa* and *Sp-PPLN2* NP genes are never co-expressed, and *Sp-FSALMFa* and *Sp-NGFFFa* NP genes are mostly co-expressed in the mouth neurons at 72hpf. Furthermore, these expression patterns vary in later larval development (1wpf)

Sp-FSALMFa* and *Sp-NGFFFa	Mouth expression					
	Only *Sp-NGFFFa*	Only *Sp-FSALMFa*	No expression	Co-expression	No co-expression	Both (Co-expression and no co-expression)
72hpf				6/8	1/8	1/8
1wpf	2/7				3/7	2/7

Sp-AN* and *Sp-Np18	Mouth expression					
	Only *Sp-AN*	Only *Sp-Np18*	No expression	Co-expression	No co-expression	Both (Co-expression and no co-expression)
72hpf		4/7		3/7		
1wpf		2/7		4/7		1/7

Sp-PPLN2* and *Sp-Np18	Mouth expression					
	Only *Sp-PPLN2*	Only *Sp-Np18*	No expression	Co-expression	No co-expression	Both (Co-expression and no co-expression)
72hpf	1/11		4/11	4/11		2/11
1wpf	3/9			3/9	2/9	1/9

Sp-FSALMFa and Sp-PPLN2	Mouth expression					
	Only *Sp-PPLN2*	Only *Sp-FSALMFa*	No expression	Co-expression	No co-expression	Both (Co-expression and no co-expression)
72hpf		2/3			1/3	
1wpf					1/2	1/2

Supplementary Table 5. An overview of co-expression profiles for six NP genes throughout all regions in the pluteus larvae.

<table>
<tr><td colspan="5">Sp-AN</td></tr>
<tr><td>Sp-PPLN2
++</td><td>Sp-Np18
++</td><td>Sp-Trh
-</td><td>Sp-FSALMFa
+</td><td>Sp-NGFFFa
+</td></tr>
<tr><td colspan="5">Sp-PPLN2</td></tr>
<tr><td>Sp-AN
++</td><td>Sp-Np18
++</td><td>Sp-Trh
-</td><td>Sp-FSALMFa
++</td><td>Sp-NGFFFa
++</td></tr>
<tr><td colspan="5">Sp-Np18</td></tr>
<tr><td>Sp-AN
++</td><td>Sp-PPLN2
++</td><td>Sp-Trh
-</td><td>Sp-FSALMFa
+</td><td>Sp-NGFFFa
+</td></tr>
<tr><td colspan="5">Sp-Trh</td></tr>
<tr><td>Sp-AN
-</td><td>Sp-PPLN2
-</td><td>Sp-Np18
-</td><td>Sp-FSALMFa
+++</td><td>Sp-NGFFFa
-</td></tr>
<tr><td colspan="5">Sp-FSALMFa</td></tr>
<tr><td>Sp-AN
+</td><td>Sp-PPLN2
++</td><td>Sp-Np18
+</td><td>Sp-Trh
++</td><td>Sp-NGFFFa
++</td></tr>
<tr><td colspan="5">Sp-NGFFFa</td></tr>
<tr><td>Sp-AN
+</td><td>Sp-PPLN2
++</td><td>Sp-Np18
+</td><td>Sp-Trh
-</td><td>Sp-FSALMFa
++</td></tr>
</table>

Key

Complete absence (-)

Rarely co-expressed 20% (+)

Sometimes co-expressed 50% (++)

Always co-expression 100% (+++)

Extended Methods

1.1 Whole mount *In situ* hybridization (ISH): detailed protocols

The protocols for chromogenic and fluorescent *in situ* hybridization (C-ISH and F-ISH) described here have been adapted from (Minokawa *et al.,*, 2004; Andrikou *et al.,*, 2013; Cole., 2009; Croce & McClay, 2010))

In all dataset, embryos and larvae at different stages were fixed overnight at 4°C in 4% PFA (Electron Microscopy Sciences), 32.5mM MOPS pH 7, 162.5 mM NaCl and 32.5% of filtered sea water; washed several times in MOPS buffer (0.1M MOPS; 0.5M Na Cl and 0.1% Tween-20) and then stored in 70% EtOH at -20 ^{o}C.

For **double F-ISH**, the embryos were first rehydrated with graded ethanol washes (70%, 50% and 30%), then washed several times in TBST (0.2M Tris pH 7.5, 0.15M NaCl, 0.1% TweenO20) at room temperature (RT), then incubated for 1 hour in hybridization buffer (Hyb: 50% deionized formamide, 10% PEG, 0.6M NaCl, 0.02M Tris pH 7.5, 0.5mg/ml yeast tRNA, 1X Denhardt's solution, 0.1%Tween-20, 5mM EDTA) at 60-65°C, finally they were incubated overnight at 60-65°C in Hyb with 0.03-0.05ng/µL of antisense DIG and DNP labelled probes. To remove excess of probe the hybridized embryos were washed in a 1:1 ratio of TBST:hybridisation buffer at 60 - 65°C), then washed four times in TBST at 60-65°C. This was followed by two washes in 1X SSC and then a single wash in 0.1X SSC at the same temperature. The embryos were then re-equilibrated in TBST at room temperature (2X washes) and probe detection was carried out with the Tyramide Signal Amplification (TSA) Systems (Perkin Elmer) using antibodies conjugated with peroxidase (POD). ISH probes were detected singularly and sequentially. Embryos were incubated with 1:2000 dilution of Anti-DIG-POD Fab fragments (Roche) or Anti-FLUO-POD, Fab fragments (Roche) or Anti-DNP horseradish peroxidase (Perkin Elmer) in Perkin Elmer blocking buffer (PERB-0.5M in TBST according to the manufacturer's instructions) for 1 hour at RT for anti-DIG antibodies, and overnight at RT for anti-DNP antibody. Embryos were washed several times at RT with TBST, and then incubated in amplification wash diluent (TBST, 0.0015% H_2O_2) for 30 min at room temperature. Embryos were then stained with 1X amplification diluent containing 1:400 dilution of Cy3 or, for 45 min or with 1X amplification diluent containing 1:400 dilution of Cy5, for 90 min. After washing with TBST to remove background staining, the horseradish peroxidase activity had to be completely eliminated to allow the second staining. For this purpose, the embryos were washed once in 1% H_2O_2, once in TBST, then once in glycine solution (0.1M glycine hydrochloride pH2.2, 0.1% Tween-20) and then washed three times with TBST. Embryos were then blocked in PERB and incubated overnight, with the second antibody as described above.

To visualize serotonergic neurons and ciliated structures in hybridized embryos, once TSA detection was completed, embryos were washed three times with PBST (137 mM NaCl, 2.7 mM KCl, 1.5 mM KH_2PO_4, 6.5 mM Na_2HPO_4, pH7.4, 0.02% (v/v) Tween) and incubated in blocking buffer (PBST, 2.5% BSA) for 30 minutes at room temperature. Embryos were then incubated for 1 hour at room temperature with anti-serotonin produced in rabbit and anti-acetylated tubulin produced in mouse (Sigma) diluted 1:500 in PBST, washed several times in PBST at RT, then incubated for 1 hour at RT in PBST containing 1:250 Alexa 488 goat anti-rabbit and Alexa 622 donkey anti-mouse (Thermo Scientifics).

Single C-ISH was conducted as described above with the following modifications. All washes were done in 1X MABT (0.1M Maleic acid pH7.5, 0.15M NaCl and 0.1% Tween-20). Embryos were

incubated in hybridization buffer with DIG-antisense labeled probes at 60-65°C for a minimum of three days to a maximum of a week. Post hybridization washes were done at the hybridization temperature, one wash with half fresh hybridization buffer and half MABT, two washes in 1X MABT for 10 minutes, one wash in 1X MABT for 30 minutes and one wash in 0.1X MABT for 30 minutes. Anti-DIG-AP Fab fragments (Roche) were used at the dilution of 1:2000. The excess of antibody was removed by washing the embryos several times with 1X MABT at room temperature for 30 minutes. Embryos were then washed twice with alkaline phosphatase buffer (0.1M Tris pH9.5, 50mM $MgCl_2$, 0.1M NaCl, 1mM Levamisole) for 30 minutes at room temperature. At this point embryos were stained in 500 µl of staining buffer (10% dimethyl formamide, 0.1M Tris pH9.5, 50mM $MgCl_2$, 0.1M NaCl, 1mM Levamisole) containing 4 µl of NBT/BCIP ready mix solution (Roche). Staining was developed in the dark, at room temperature and monitored under dissecting microscope. When a suitable level of staining had developed, the staining reaction was stopped by washing few times in 1X MABT containing 0.05M EDTA and transferred into 50% glycerol and stored at 4°C indefinitely.

1.2 Immunostaining

Embryo and larvae at the desired stage were fixed in 4% paraformaldehyde (Electron Microscopy Sciences) in PBS or PEM buffer (Vielkind & Swierenga, 1989), at room temperature for 5-15 minutes and then washed three times in PBS. For Serotonin and Acetylated Tubulin, samples were washed once in PBSTx (0.01% Triton x-100) for 20 minutes, then twice in PBST. For SynaptotagminB the samples had an extra step with 100% methanol for two minutes on ice, followed by two washed in PBS and two in PBST.

Before incubation with primary antibody, samples were incubated in blocking buffer (4% goat serum in PBST) for 30min at RT. Rabbit polyclonal anti-serotonin (Sigma) and mouse anti-acetylated tubulin (Sigma) were both used at a dilution of 1:500. Mouse anti-synaptotagminB (SynB/1E11) (Nakajima *et al.,* 2004) was used at a dilution of 1:5 diluted in blocking buffer and incubated overnight at 4°C. Excess of antibody was washed in PBT at RT for several times. Secondary antibodies Alexa 488 goat anti-rabbit, Alexa 633 donkey anti-mouse and Alexa 488 goat anti-mouse (Thermo Scientifics) were used at a dilution of 1:250 in blocking buffer.

1.3 EdU labelling

Larvae at four days of development were incubated with a 10mM stock solution of ethynyl deoxyuridine (EdU) for two hours at room temperature. Larvae were fixed in 4% paraformaldehyde in PEM buffer and washed as follows:

- Three times in PBST (0.1% Tween).
- One time in PBSTx (0.01% Triton x-100) for 45 minutes.
- Two washes in PBST.
- One wash in 100% methanol for two minutes on ice,
- Twice with PBS
- Twice with PBST.
- The Click-iT reaction mixture (Click-iT™ EdU Alexa Fluor™ 555 Imaging Kit, Thermo Fisher) was added to the larvae and incubated for 30 minutes at room temperature.
- The larvae were then washed with the Click-iT reaction rinse buffer for 30 minutes
- Washed twice with PBST.

- Nuclear stains were obtained as described in the main text.

Immunolabeling was conducted as described above either before or after the Click-iT reaction.

1.4 Quantitative PCR (QPCR)

Total RNA was isolated from batches of sea urchin embryos, larvae and juveniles at different stages. The RNA was extracted using the RNAeasy Micro Kit (Qiagen) according to manufacturer's instructions. First-strand cDNA was synthesised using a maximum of 1µg of total RNA and the iScript™ cDNA synthesis kit (Bio-Rad), as described by the manufacturer. The cDNA was diluted to 2.8ng/µl (corresponding to 1 embryo/µl) and used directly for quantitative PCR (QPCR) analysis. QPCR was conducted as previously described (Rast *et al.,* 2000) and each combination of cDNA and primers was run in quadruplicates on a 384-well plate on a QuantStudio™ 6 Flex Real-Time PCR System (ThermoFisher Scientific). Each reaction mix (final volume of 9 µl) contained the following: 0.5µl of cDNA (2.8ng/µl), 4.5µl 2X Power SYBR green PCR Master Mix (ThermoFisher Scientific) and 0.55µl of each primer (2.5pmole/µl) and run in a two step PCR for 40 cycles. Dissociation curve protocol has been introduced in every run as a quality control of the amplified product.

For embryonic stages (24-70hpf) *Sp-ubq* (Nemer *et al.,* 1991) was used in each plate as internal standard, while for larval stages 18S (Ransick *et al.,* 2002) was used, which are known to remain relatively constant during development (Nemer *et al.,* 1991; Oliveri and Davidson 2004; Ransick *et al.,* 2002). To calculate the level of expression of each gene at each embryonic stage we used the strategy described in Oliveri and Davidson 2004. We first average the cycles at the threshold (Ct) for the quadruplicates and calculate the standard deviation (Stdev; Supplementary Figure 1 and 2), we then normalised the data against the *Sp-ubq* average Ct (or 18S) of the plate. To calculate the folds of difference relative to *Sp-ubq* expression we used as factor the average of amplification efficiency used in Oliveri and Davidson 2004. Absolute numbers of transcripts per embryo were calculated based on the publicly available quantitative transcriptome data (echinobase) for various genes (Supplementary Figure 4A) and identifying an average conversion factor. The relative expression is calculated by identifying the maximum level of expression for the entire developmental series of each gene. The primers used for the QPCR experiments can be found in Supplementary Table 2.

Supplementary References

Andrikou, C., Iovene, E., Rizzo, F., Oliveri, P., and Arnone, M. I. (2013). Myogenesis in the sea urchin embryo: the molecular fingerprint of the myoblast precursors. *Evodevo* 4, 1–16. doi:10.1186/2041-9139-4-33.

Cole, A. G., Rizzo, F., Martinez, P., Fernandez-Serra, M., and Arnone, M. I. (2009). Two ParaHox genes , SpLox and SpCdx , interact to partition the posterior endoderm in the formation of a functional gut. *Development* 136, 541–549. doi:10.1242/dev.029959.

Croce, J. C., and McClay, D. R. (2010). Dynamics of Delta / Notch signaling on endomesoderm segregation in the sea urchin embryo. *Development* 137, 83–91. doi:10.1242/dev.044149.

Minokawa, T., Rast, J. P., Arenas-mena, C., Franco, C. B., and Davidson, E. H. (2004). Expression

patterns of four different regulatory genes that function during sea urchin development. *Gene Expr. Patterns* 4, 449–456. doi:10.1016/j.modgep.2004.01.009.

Nakajima, Y., Kaneko, H., Murray, G., and Burke, R. D. (2004). Divergent patterns of neural development in larval echinoids and asteroids. *Evol. Dev.* 6, 95–104.

Nemer, M., Rondinelli, E., Infante, D., and Infante, A. A. (1991). Polyubiquitin RNA Characteristics and Conditional Induction in Sea Urchin Embryos. *Dev. Biol.* 145, 255–265.

Oliveri, P., and Davidson, E. H. (2004). Gene Regulatory Network Analysis in Sea Urchin Embryos. *Methods Cell Biol.* 74, 775–794.

Ransick, A., Rast, J. P., Minokawa, T., Calestani, C., and Davidson, E. H. (2002). New Early Zygotic Regulators Expressed in Endomesoderm of Sea Urchin Embryos Discovered by Differential Array Hybridization. *Dev. Biol.* 246, 132–147.

Rast, J. P., Amore, G., Calestani, C., Livi, C. B., Ransick, A., and Davidson, E. H. (2000). Recovery of Developmentally Define Gene Sets from High-Density cDNA Macroarrays. *Dev. Biol.* 228, 270–286.

Vielkind, U., and Swierenga, S. H. (1989). A simple fixation procedure for immunofluorescent detection of different cytoskeletal components within the same cell. *Histochemistry* 91, 81–88.
